# Serotype and Genotype Distribution among Invasive *Streptococcus pneumoniae* Isolates in Colombia, 2005–2010

**DOI:** 10.1371/journal.pone.0084993

**Published:** 2014-01-08

**Authors:** Eliana L. Parra, Viviana Ramos, Olga Sanabria, Jaime Moreno

**Affiliations:** Grupo de Microbiología, Instituto Nacional de Salud, Bogotá, Colombia; Rockefeller University, United States of America

## Abstract

In Colombia, a laboratory-based surveillance of invasive *Streptococcus pneumoniae* isolates as part of SIREVA II PAHO has been conducted since 1994. This study describes the serotype distribution, antimicrobial resistance, and genetic relationships of pneumococcal isolates recovered in Colombia from 2005 to 2010. In this study, demographic data of invasive *S. pneumoniae* isolates were analyzed, and antimicrobial susceptibility patterns were determined. Pulse field gel electrophoresis (n = 629) and multilocus sequence typing (n = 10) were used to determine genetic relationship of isolates with minimal inhibitory concentration to penicillin ≥0.125 µg/mL. A total of 1775 isolates of *S. pneumoniae* were obtained. Fifteen serotypes accounted for 80.7% of isolates. Serotype 14 (23.1%) was the most frequent in the general population. Penicillin resistance was 30.7% in meningitis and 9.0% in non-meningitis. Clones Spain^6B^ST90, Spain^9V^ST156, Spain^23F^ST81, and Colombia^23F^ST338 were associated to isolates. Additionally, serotype 6A isolates were associated with ST460 and ST473, and 19A isolates with ST276, ST320, and ST1118. In conclusion, the surveillance program provided updated information of trends in serotype distribution, antimicrobial resistance and the circulation of clones in invasive pneumococcal diseases. These results could be helpful to understand the epidemiology of *S. pneumoniae* in Colombia, and provide a baseline to measure the impact of vaccine introduction.

## Introduction

*Streptococcus pneumoniae* is an important cause of invasive pneumococcal disease (IPD), as pneumonia, meningitis, and sepsis [1]. At present, over 95 serotypes have been described [2]–[5], which present variation in IPD, age groups, virulence and geographical distribution [6]. Serotype distribution has changed in countries that have implemented pneumococcal conjugate vaccines (PCV), as PCV7 (serotypes 4, 6B, 9V, 14, 18C, 19F, and 23F), and currently available vaccines, PCV10 (PVC7 serotypes plus serotypes 1, 5, 7F) and PCV13 (PCV10 serotypes plus serotypes 3, 6A, 19A). Although vaccine serotypes have declined after PCVs introduction, non-PCV serotypes have increased in IPD [7]–[9]. Additionally, for over three decades antibiotic-resistant strains of *S. pneumoniae* have increased worldwide [9], which has been related with the spread or emergence of clones [10]. In 2009, PCV7 universal vaccination started in children <2 years of age with a 2+1 dose schedule in 12 regions of Colombia, including Bogotá. Subsequently, PCV7 was switched to PCV10 in 2010, and extended to the whole country [11], [12].

A laboratory-based passive surveillance of invasive *S. pneumoniae* isolates as part of SIREVA II PAHO has been conducted in Colombia, since 1994 [13]. The aim of this study was to determine serotype distribution, antimicrobial susceptibility and genetic relationships in Colombian pneumococcal isolates recovered from 2005 to 2010.

## Materials and Methods

### *S. pneumoniae* Isolates

Invasive isolates studied were received at the Instituto Nacional de Salud in Colombia as part of a passive surveillance, through the IPD National Surveillance (SIREVA II– program) from January 2005 to December 2010. A case of IPD was defined as isolation of *S. pneumoniae* from a normally sterile body site such as blood, cerebrospinal fluid or other normally sterile body fluid. The isolates were identified by morphology, optochin sensitivity and bile solubility. The present study includes 395 invasive isolates from children <2 years, which were previously studied and published by Parra et al. [14].

### Serotyping and Antibiotic Susceptibility Testing

Serotype was determined by Quellung reaction. Antimicrobial susceptibility tests were performed by Kirby-Bauer disk diffusion and the broth micro-dilution methods to penicillin (PEN), ceftriaxone (CRO), trimethoprim-sulfamethoxazole (SXT), chloramphenicol (CHL), tetracycline (TET), erythromycin (ERY) and vancomycin (V). The Clinical and Laboratory Standards Institute (CLSI) guidelines were used [15]. Resistance to penicillin and ceftriaxone was analyzed by diagnosis of meningitis and non-meningitis. Multi drug resistance (MDR) was defined as resistant to three or more antibiotic classes.

### Molecular Characterization

Isolates with minimal inhibitory concentration (MIC) to penicillin ≥0.125 µg/mL, were characterized by pulsed-field gel electrophoresis (PFGE), according to a previous report [16]. R6 strain and representative strains of clones Spain^9V^ST156, Spain^23F^ST81, Spain^6B^ST90 and Colombia^23F^ST338 were used as reference. PFGE patters were analyzed with the Gelcompare II software (Copyright Applied Maths 1998–2005). A dendrogram was constructed by the unweighted-pair group method with arithmetic means (UPGMA), using the Dice similarity coefficient, an optimization value of 1.5% and a tolerance position of 1.3%. Clusters of PFGE patterns exhibiting similarity of >75% were designated by capital letters. Sequence type (ST) from representative isolates in each clonal group was determined according to Enright et al [17]. Isolates for MLST were selected according the PFGE clusters and serotype. Software available on the MLST Web site was used to analyze genetic profiles [18].

### Statistical Analysis

Analysis data of serotypes and IPD were stratified by age groups (<2, 2 to <5, 5 to 14, 15 to 29, 30 to 49, 50 to 64 and ≥65 years). Data were analyzed using Microsoft Excel™ and Statistical Package for Social Sciences (SPSS) software® (version 18). Statistical significance differences were assessed using Chi-square test with a significance level of <0.05.

## Results

A total of 1775 *S. pneumoniae* invasive isolates were collected by 26 Public Health Laboratories and the Capital District. Ten Political Administrative Divisions of Colombia provided 95.4% of isolates (Figure S1). Among 1775 isolates, 59.4% were from male patients. Isolates were recovered more frequently in children <2 years (30.8%) of age and adults ≥50 (22.8%) (Table 1).

**Table 1:** General distribution of *S. pneumoniae* isolates by age group recovered in Colombia from 2005 to 2010.

Year	Age group in years							
	<2	2 to <5	5 to 14	15 to 29	30 to 49	50 to 64	≥65	Total
	n (%)							
2005	85(30.6)	30(10.8)	29(10.4)	28(10.1)	46(16.5)	23(8.3)	37(13.3)	278
2006	104(35.3)	34(11.5)	33(11.2)	23(7.8)	43(14.6)	26(8.8)	32(10.8)	295
2007	110(32.8)	37(11.0)	32(9.6)	28(8.4)	48(14.3)	39(11.6)	41(12.2)	335
2008	100(38.8)	34(13.2)	23(8.9)	17(6.6)	36(14.0)	24(9.3)	24(9.3)	258
2009	68(25.9)	35(13.3)	39(14.8)	21(8.0)	38(14.4)	31(11.8)	31(11.8)	263
2010	80(23.1)	46(13.3)	40(11.6)	38(11.0)	45(13.0)	47(13.6)	50(14.5)	346
Total	547(30.8)	216(12.2)	196(11.0)	155(8.7)	256(14.4)	190(10.7)	215(12.1)	1775

Isolates were recovered from blood (70.2%), cerebrospinal fluid (23.8%) and others (6.0%). Pneumonia (37.6%), meningitis (26%), and sepsis (24%) were the most common diagnoses. Pneumonia was the most frequent diagnosis in all age groups. In the <2 years group meningitis (31.8%) was the second diagnosis more frequent, while in the 2 to <5 and ≥50 age groups, sepsis was the second more important diagnosis with a percentage of 20.8% and 30.1%, respectively (Table 2).

**Table 2:** General distribution of *S. pneumoniae* isolates by diagnosis and age group.

Diagnosis	Age group							Total
	<2	2 to <5	5 to 14	15 to 29	30 to 49	50 to 64	≥65	
Pneumonia	192(35.1)	107(49.5)	65(33.2)	55(35.5)	91(35.5)	63(33.2)	95(44.2)	668(37.6)
Meningitis	174(31.8)	23(10.6)	60(30.6)	51(32.9)	81(31.6)	47(24.7)	27(12.6)	463(26.1)
Sepsis	114(20.8)	60(27.8)	43(21.9)	35(22.6)	57(22.3)	56(29.5)	66(30.7)	431(24.3)
Others	67(12.2)	26(12.0)	28(14.3)	14(9.0)	27(10.5)	24(12.6)	27(12.6)	213(12.0)
Total	547	216	196	155	256	190	215	1775

Others included febrile syndrome n = 88 (5%), bacteremia n = 30 (1.7%), and without a specific pneumococcal diagnosis, but recovered by blood culture n = 99 (5.6%).

### *S. pneumoniae* Serotype Distribution

**Figure 1:**
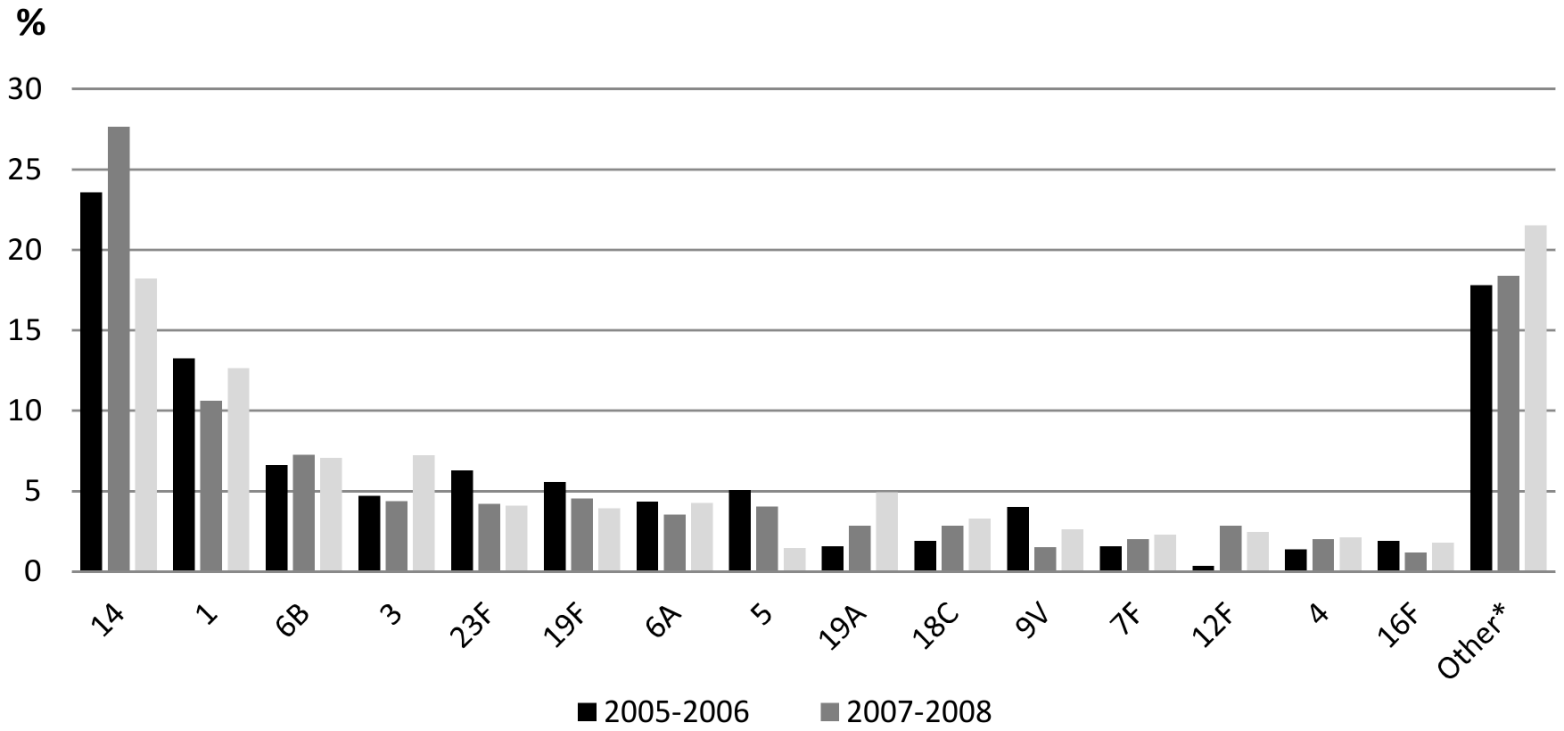
Annual frequency of serotypes recovered in Colombia from 2005 to 2010. *In other were included 41 serotypes with a frequency less than 1.1%. The number and frequency of all isolates are shown in the Table S1.

A total of fifty-six serotypes were identified, and fifteen serotypes accounted for 80.7% of the total isolates: 14 (23.1%), 1 (12.2%), 6B (7%), 3 (5.5%), 23F (4.8%), 19F (4.7%), 6A (4.1%), 5 (3.5%), 19A (3.2%), 18C (2.7%), 9V (2.7%), 7F (2.0%),12F (1.9%), 4 (1.9%), and 16F (1.6%). The other serotypes presented a proportion of 19.3% (Table S1). Figure 1 shows the frequency of these serotypes in 2005–2006, 2007–2008 and 2009–2010. Among serotypes that presented a significant decrease from 2007–2008 to 2009–2010 were serotype 14, which changed from 27.7% to 18.2% (p<0.001) and serotype 5 from 4.0% to 1.5% (p<0.001). In this same period serotypes 3 and 19A increased from 4.4% to 7.2% (p = 0.01) and 2.9% to 4.9% (p = 0.03), respectively. The other serotypes changed from 17.8% in 2005–2006 to 21.5% in 2009–2010 (p = 0.05) (Figure 1).

Serotype 14 was the most prevalent in <2 and 2 to <5 age groups. Serotype 1 was mainly observed in the 2 to 5 (15.7%), 5 to 14 (23.5%), 15 to 29 (15.2%) 30 to 49 age groups. Serotype 3 was the most frequent in adults ≥50 (11.9%) (Table 3). Rank order of the most common serotypes from patients with pneumonia were 14 (28.3%), 1 (15.1%), 3 (6.0%), 6B (5.4%), 5 (5.1%) and 19A (3.6%); whereas, in meningitis the most frequent serotypes were 14 (15.3%), 6B (10.2%), 23F (8.0%), 19F (7.6%), 1 (6.7%) and 6A (5.8%) (Table S2).

**Table 3:** General distribution of serotypes by age group recovered in Colombia, 2005–2010.

Serotypes	Age groups in years							Total
	<2	2 to <5	5 to 14	15 to 29	30 to 49	50 to 64	≥65	
14	197(36.0)	87(40.3)	25(12.8)	25(16.1)	35(13.7)	19(10.0)	22(10.2)	410
1	42(7.7)	34(15.7)	46(23.5)	24(15.5)	34(13.3)	23(12.1)	13(6.0)	216
6B	60(11.0)	11(5.1)	12(6.1)	8(5.2)	12(4.7)	7(3.7)	14(6.5)	124
3	15(2.7)	6(2.8)	9(4.6)	7(4.5)	12(4.7)	21(11.1)	27(12.6)	97
23F	21(3.8)	12(5.6)	10(5.1)	10(6.5)	12(4.7)	9(4.7)	12(5.6)	86
19F	29(5.3)	7(3.2)	11(5.6)	7(4.5)	13(5.1)	10(5.3)	6(2.8)	83
6A	24(4.4)	5(2.3)	13(6.6)	4(2.6)	7(2.7)	7(3.7)	12(5.6)	72
5	12(2.2)	6(2.8)	5(2.6)	6(3.9)	12(4.7)	9(4.7)	12(5.6)	62
19A	18(3.3)	18(8.3)	3(1.5)	2(1.3)	8(3.1)	3(1.6)	4(1.9)	56
18C	25(4.6)	5(2.3)	6(3.1)	4(2.6)	4(1.6)	2(1.1)	2(0.9)	48
9V	15(2.7)	4(1.9)	7(3.6)	4(2.6)	8(3.1)	4(2.1)	6(2.8)	48
7F	5(0.9)	2(0.9)	2(1.0)	7(4.5)	9(3.5)	3(1.6)	7(3.3)	35
12F	5(0.9)	0(0.0)	0(0.0)	1(0.6)	9(3.5)	11(5.8)	8(3.7)	34
4	8(1.5)	0(0.0)	0(0.0)	4(2.6)	7(2.7)	7(3.7)	7(3.5)	33
16F	5(0.9)	4(1.9)	2(1.0)	3(1.9)	4(1.6)	5(2.8)	6(2.7)	29
Others*	66(12.1)	15(6.9)	45(23.0)	39(25.2)	70(27.3)	50(26.3)	57(26.4)	342
Total	547(30.8)	216(12.2)	196(11.0)	155(8.7)	256(14.4)	190(10.8)	215(12.1)	1775

Others included a total of 41 serotypes, frequency less to 1.1%.

**Figure 2:**
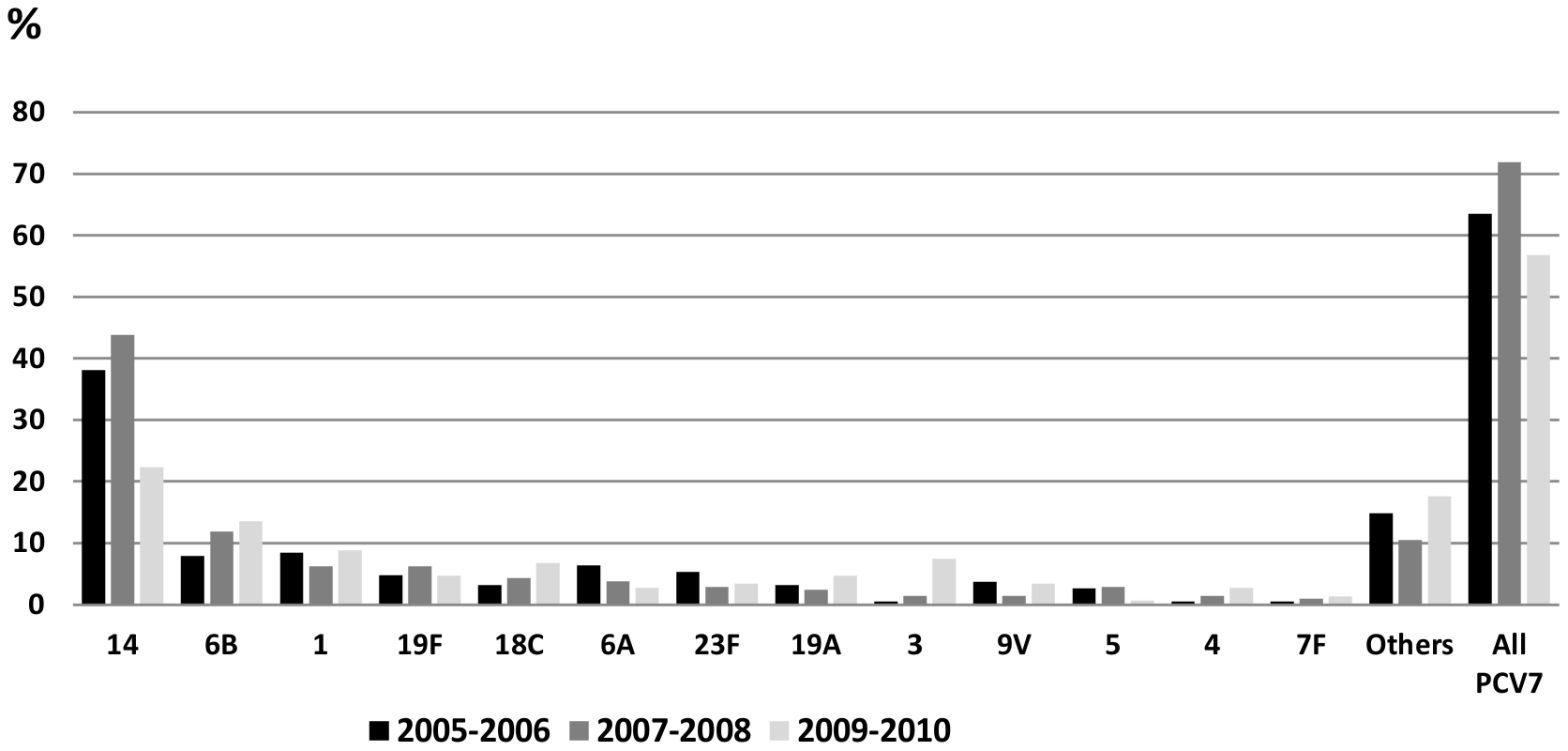
Serotypes frequency in children <2 years old, 2005–2010.

Isolate distribution in children <2 years old presented a frequency of 38.8% in 2008, and it decreased to 25.9% and 23.1% in 2009 and 2010, respectively (Table 1). Additionally, in this group the frequency of PCV7 serotypes changed from 71.9% in 2007–2008 to 56.8% in 2009–2010 (p<0.001). This change was mainly due to a decrease in serotype 14, which changed from 43.8% (2007–2008) to 22.3% (2009–2010) (p<0.001). PCV10 and PCV13 frequency serotypes were 75.7% and 86.1%, respectively. Serotypes 1 and 3 increased from 6.2% to 8.8% (p = 0.18), and from 1% to 7.4% (p<0.001), respectively (Figure 2).

### Penicillin and Ceftriaxone Susceptibility

Penicillin resistance was 30.7% (MIC_50/90_ 0.03/2.0 µg/mL) in meningitis, and 9.0% in non-meningitis (intermediate 8.6% and high 0.4%; MIC_50/90_ of 0.03/2.0 µg/mL). Resistance to ceftriaxone was 15.7% in meningitis (intermediate 12.5% and high 3.2%) and 8.5% in non-meningitis (Table S3). Serotypes related with resistance to penicillin were 14 (60.0%), 23F (10.0%), 6B (6.5%), 19A (6.5%), 9V (4.2%), 6A (3.5%), 19F (3.5%) and other serotypes (n = 13) (5.8%).

### Susceptibility to other Antimicrobials

Isolates showed resistance to SXT (43.8%), TET (17.1%), ERY (6.9%) and CHL (3.7%). All isolates were susceptible to vancomycin (Table S3). Resistance to ERY increased from 7.6% (2009) to 9.8% (2010) (p = 0.17), associated to serotypes 6A (21.1%), 19A (20.3%), 6B (19.5%), 14 (13.0%) and other serotypes (n = 12) (26.1%). The overall rate of MDR was 7.3% (n = 130 isolates), 9.7% in meningitis and 6.4% in non-meningitis. The most common MDR patterns were PEN-SXT-TET (16.2%), followed by PEN-CRO-SXT-ERY-TET (13%) and CHL-SXT-TET (10.8%).

### Molecular Characterization

From a total of 629 isolates (MIC PEN ≥0.125 µg/mL) typed by PFGE, 85.4% belonged to one of 11 clusters (A–K) (Table 4 and figure S2). Cluster A, grouped mainly isolates 23F related to the Spain^23F^ST81 clone, and serotypes 19A and 19F. Except for the 19A isolate, all isolates were MDR. Cluster B presented relation to Spain^9V^ST156 clone, which grouped serotypes 9V, 14, 19A and 23F and MDR was found in 18 (4.8%) isolates. Cluster C related to Colombia^23F^ST338 was associated mainly with isolates serotype 23F. This cluster also associated serotype 19A (a single locus variant of ST338), 19F, 23A, 23B, and MDR was observed in 21.2% (n = 7) of isolates. In cluster D, only serotype 6B isolates associated with Spain^6B^ST90 clone were observed, which presented MDR of 78.3%. In clusters E, F and G, formed by serotypes 19A, related to ST320, ST1118 and ST276, respectively, all isolates associated to ST320 and ST276 were MDR, and in isolates grouped to ST1118 MDR was not observed. Clusters H and I included serotype 6A isolates. Cluster H, conformed by PEN and ERY resistant isolates were related to ST473, and cluster I, PEN and SXT resistant isolates associated to ST460. Clusters J and K grouped mainly with isolates serotype 14 (Table 4).

**Table 4:** Serotype distribution and genetic association in isolates with MIC values ≥0.125 (*n = *629).

PFGE patterns	Clone and ST	Serotypes								
		14	23F	6B	19A	9V	6A	19F	Other***	Total
A	Spain^23F^ST81	0	12	0	1	0	0	1	0	14
B	Spain^9V^ST156	348	1	0	2*	30	0	0	0	381
C	Colombia^23F^ST338	0	27	0	1	0	0	3	2	33
D	Spain^6B^ST90	0	0	23	0	0	0	0	0	23
E	ST320	0	0	0	8	0	0	2	0	10
F	ST1118	0	0	0	6	0	0	0	1	7
G	ST276	0	0	0	10	0	0	0	0	10
H	ST473	1	0	0	0	0	15	0	1	17
I	ST460	2	0	0	0	0	10	0	3	15
J	–	9	0	0	0	0	0	0	0	9
K	–	13	0	0	0	0	0	1	4	18
NR**	–	12	10	18	9	2	7	13	21	92
Total		385	50	41	37	32	32	20	32	629

One isolate was confirmed as ST156.

NR: unrelated isolates in specific cluster groups among isolates into dendrogram.

Other: the dendrogram shows these serotypes.

Note: the dendrogram of isolates is shown in the figure S2.

## Discussion

This study described serotype distribution, antimicrobial resistance and circulation of clones associated with penicillin non-susceptibility, in invasive *S. pneumoniae* isolates recovered in Colombia from 2005 to 2010. The result showed that pneumonia and meningitis were the most frequent IPD. Both diagnoses have been reported as the most frequent in Colombia [13], as well as in Latin America and the Caribbean region, with an annual burden of 327,000 pneumonia cases, nearly 4,000 cases of meningitis and 1,229 cases of pneumococcal sepsis [19]. Even though sepsis was the second most common diagnosis in 2–6 and ≥50 years age patient, this could be due to variations in clinical definition of syndromes informed in the pneumococcal surveillance, where pneumococcal community-acquired pneumonia and meningitis are leading causes of sepsis [20], [21].

In general, serotypes 14, 1, 6B, 23F, 19F and 6A were the most frequent, similar to a study published previously in Colombia [13] and worldwide [22], [23]. Serotype 14 was the most prevalent in the national surveillance, and it was the main cause of IPD in children <5 years age. In a systematic review, realized to estimate the global and regional distributions of serotypes causing IPD in children <5 years of age, serotype 14 was the most common serotype accounting for 12%–29% of IPD worldwide [23]. Serotype 1 was a frequent cause of IPD in older children; this serotype has a lower propensity to cause infections in the older age groups [24]. An increase in serotype 3 frequency was observed, which was mainly found in the ≥50 age group. This serotype has been associated with a relative risk of infection among middle-aged people up to a peak in older adults [25].

Serotype 14 continues as the main cause of IPD and most isolates were associated with Spain^9V^ST156 clone, similar to a previous report [13]. This clone is resistant to PEN and SXT, and has become widely disseminated among invasive isolates [26]. Furthermore, serotype 19A and 19F variants were observed, which have already been identified in others studies, suggesting a high tendency of this clonal cluster to undergo capsular switching events [26]. Isolates serotype 23F were related genetically to Spain^23F^ST81 clone; this clone presents resistance pattern to PEN, CHL, TET, and many isolates have additional resistance to fluoroquinolones and macrolides [27], [28]. This clone displays a high genetic variability and some isolates have switched their capsular type to 9N, 19A, 19F, 14, 15B, 3 and 6A over the last decades [29], [30]. Additionally, 23F isolates were associated to Colombia^23F^ST338, a clone initially identified in Colombian isolates [31], and also have been reported in other countries [32]–[34]. MDR serotype 6B isolates were related to Spain^6B^ST90 clone. These international clones continue circulating in Colombia and were associated with resistance to antimicrobials. The implementation of PCV may reduce the prevalence of these clones, leading to the spreading of non-vaccine clones or capsular variants of international drug-resistant clones [34], [35], and continuous surveillance will be necessary to follow future changes.

The incidence and prevalence of IPD cases caused by serotype 19A increased substantially in the post-PCV7 introduction era and has been associated with MDR [36]. During the surveillance period, serotype 19A increased from 1.5% to 4.9%. In Latin American and Caribbean countries, serotype19A remains a less common agent of IPD than other serotypes and a significant increase in frequency was noted only in Argentina and Colombia [37]. Serotype 19A isolates were found related to the ST230 and ST320. The ST230 represents the Denmark^14-^ST230 clone and is an important cause for the maintenance of antimicrobial resistance. This clone was reported in the preceding years to PCV7 introduction in Portugal [38] and has been detected in several countries, such as France and Spain [39], [40]. ST320 has become one of the major STs among 19A *S. pneumoniae* isolates in USA and Canada after the introduction of PCV7 [41] and is prevalent in several Asian countries [42]. Among serotype 6A isolates, ST473 was identified. The ST473 is a penicillin-non-susceptible and erythromycin-resistant clone which has been identified among serotypes 6A and 6C invasive and noninvasive isolates [43], [44].

Antimicrobial resistance in *S. pneumoniae* has been one of the main worldwide problems, especially penicillin and erythromycin non-susceptible isolates [9]. The rates of penicillin-resistant pneumococcal meningitis and non-meningitis in Colombia were lower than other Latin American countries such as Bolivia, Ecuador, Mexico, Peru and Venezuela [45]. In Colombia, antimicrobial resistance has been associated with the presence of multi-resistant clones [13]. The increase of non-PCV7 serotypes and clonal spread of serotype 19A (ST 230 and ST 320) and 6A (ST473) strains would be the main reasons for prevalence of erythromycin resistance in Colombia.

In 2009, PCV7 vaccination started in children less than 2 years of age in 12 regions of Colombia [11], [12]. A significant decline in the frequency of PCV7 vaccine serotypes in <2 years age group was observed, this could be associated with the PCV7 introduction in Colombia, similar to other countries where routine PCV7 use has resulted in a significant decrease or a near elimination of PCV7 serotypes in IPD for children [46], [47].

The limitations in this study were related to the fact that 81.3% of *S. pneumoniae* were sent from three Political Administrative Divisions of Colombia; therefore, the data may not reflect a national status of serotype distribution, antimicrobial resistance and genetic relations of isolates. However, the results may reflect an overall trend in the changes of *S. pneumoniae* in Colombia. Additionally, the molecular characterization was based on non-susceptible isolates and not on the total of isolates, which provide an incomplete clonal structure. Finally, serotype 6D is not differentiated from serotype 6B; thus, serotype 6D isolates could be included among serotype 6B isolates.

In conclusion, the surveillance program has been essential in assessing changes in antimicrobial resistance, serotypes and clones in resistant isolates. Continuous monitoring of IPD is necessary to measure the impact of PCV10/13 introduction and also to understand the Colombian epidemiology of *S. pneumonia.*

## Supporting Information

Figure S1 **Distribution of** ***S. pneumoniae*** **recovered from political administrative division of Colombia.**
(TIF)

Figure S2 **Genetic relationships dendrogram of** ***Streptococcus pneumoniae*** **isolates by PFGE.**
(PDF)

Table S1 **General frequency of serotypes by years of surveillance.**
(XLS)

Table S2 **Serotype distribution by diagnosis.**
(XLS)

Table S3 **Susceptibility and minimum inhibitory concentrations in *S. pneumoniae* isolates in invasive pneumococcal diseases during 2005 and 2010.**
(DOC)
