# Supplementary figures and images for: Serotype and Genotype Distribution among Invasive Streptococcus pneumoniae Isolates in Colombia, 2005–2010
Source: PLoS One. 2014 Jan 8;9(1):e84993. doi: 10.1371/journal.pone.0084993 (PMC3885649; doi:10.1371/journal.pone.0084993)

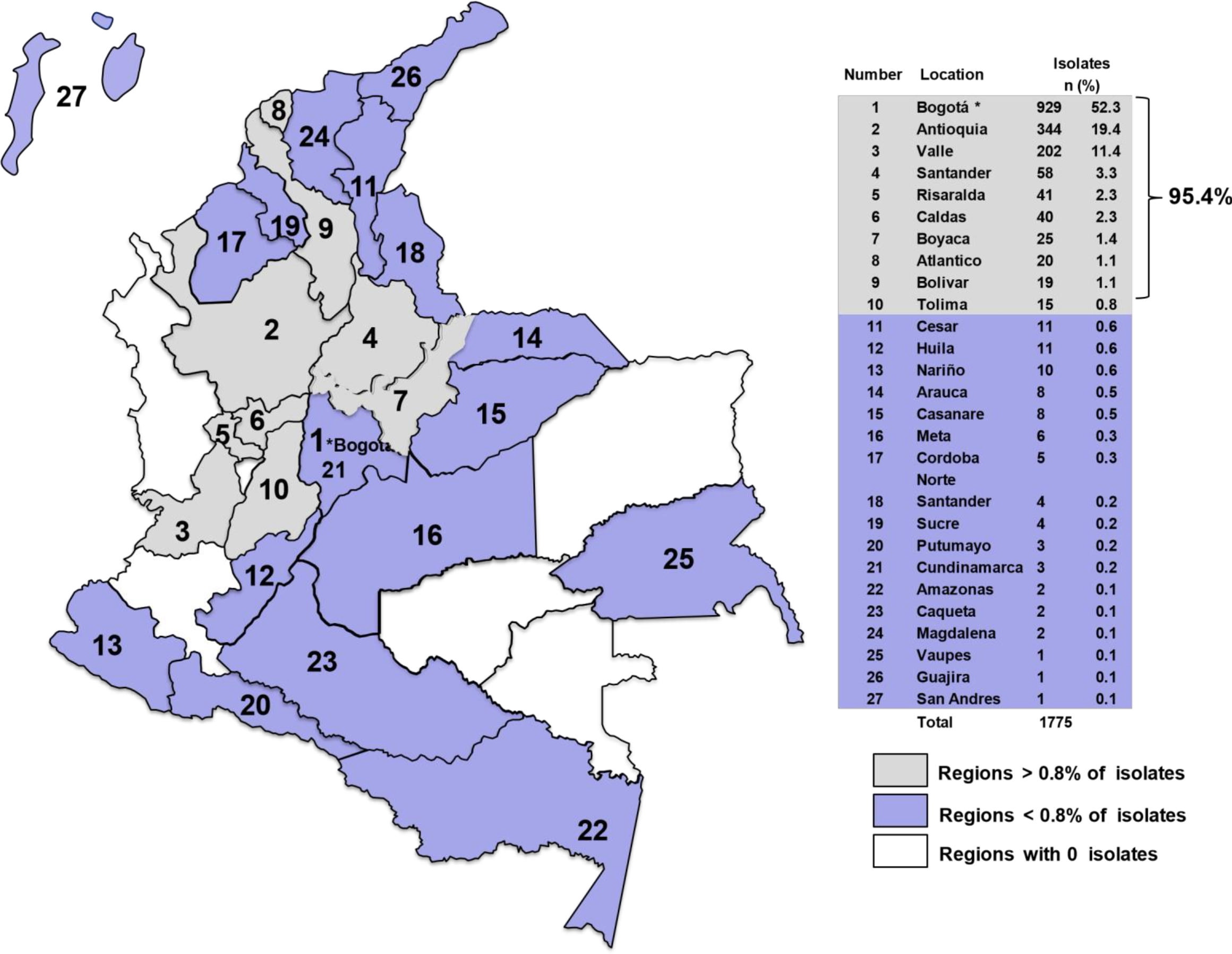

Supplement: Figure S1 — Distribution of S. pneumoniae recovered from political administrative division of Colombia. (TIF) [file pone.0084993.s001.tif]
