# Supplementary material for: Serotype and Genotype Distribution among Invasive Streptococcus pneumoniae Isolates in Colombia, 2005–2010
Source: PLoS One. 2014 Jan 8;9(1):e84993. doi: 10.1371/journal.pone.0084993 (PMC3885649; doi:10.1371/journal.pone.0084993)
Supplement: Figure S2 — Genetic relationships dendrogram of Streptococcus pneumoniae isolates by PFGE. (PDF) [file pone.0084993.s002.pdf]

Dice (Opt:1.50%) (Tol 1.3%-1.3%) (H>0.0% S>0.0%) [0.0%-100.0%]

neumococo

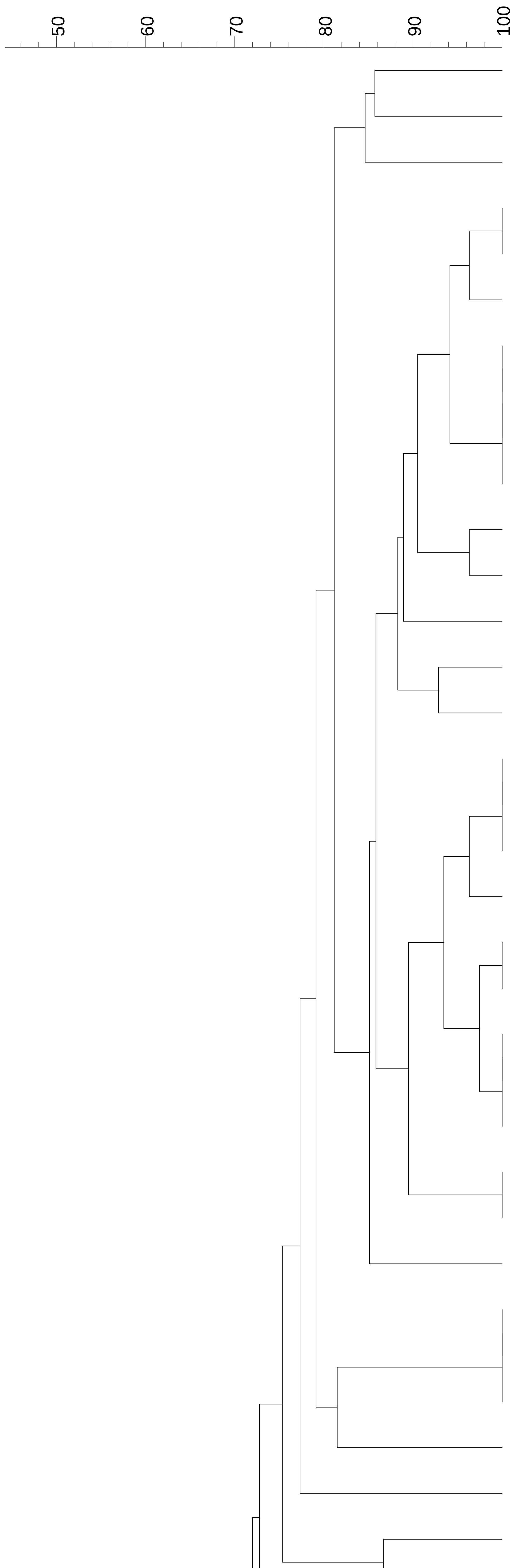

Isolates	Serotype	Cluster	Location	Year	Resistance	Diagnosis
E1695	23F	C42	BOGOTÁ	2010	PI-SXT	PNEUMONIA
4358	23F	C39	BOGOTÁ	2008	PI-SXT-TET	MENINGITIS
4888	23B	C41	BOGOTÁ	2010	PI-SXT	MENINGITIS
3035	23F	C36	ANTIOQUIA	2005	PI-SXT-TET	F. SYNDROME
4296	19F	C36	BOGOTÁ	2008	PI-SXT-TET	MENINGITIS
E1275	23F	C12	BOGOTÁ	2007	PI-SXT	PNEUMONIA
E1024	23F	C34	BOGOTÁ	2006	PI-SXT-TET	PNEUMONIA
5037	23F	C34	VALLE	2010	PI-SXT	SEPSIS
E1612	23F	C34	BOGOTÁ	2010	PI-SXT-TET	SEPSIS
E1626	23F	C34	ANTIOQUIA	2010	PI-SXT-TET	SEPSIS
4550	23F	C27	SANTANDER	2009	PI-SXT	SEPSIS
E1377	19F	C39	BOGOTÁ	2008	PI-SXT	PNEUMONIA
E1178	23F	C47	BOGOTÁ	2007	PI-SXT	PNEUMONIA
3902	23F	C49	BOGOTÁ	2007	PI-TET	PNEUMONIA
4976	23A	C44	BOGOTÁ	2010	PI	PNEUMONIA
3381	19A	C- ST338.	ATLANTICO	2006	PI-TET	MENINGITIS
Colombia23F-ST338	23F	C			P-CHL-TET-STX	
E1419	23F	C	SANTANDER	2008	PI-SXT	SEPSIS
4244	23F	C30	HUILA	2008	PI-SXT-TET	MENINGITIS
5147	23F	C32	BOGOTÁ	2010	PI-SXT	F. SYNDROME
E1115	23F	C32	ANTIOQUIA	2006	PI-SXT	MENINGITIS
3983	23F	C22	ANTIOQUIA	2007	PI-SXT	SEPSIS
E883	23F	C22	BOGOTÁ	2005	PI-SXT	BACTERIEMIA
E1561	23F	C30	ANTIOQUIA	2009	PI-SXT	SEPSIS
E991	23F	C31	SANTANDER	2005	PI-SXT	PNEUMONIA
4705	23F	C31	VALLE	2009	PI-SXT	F. SYNDROME
2917	23F	C35	VALLE	2005	PI-SXT	MENINGITIS
E1065	23F	C51	ANTIOQUIA	2006	PI-SXT-TET	MENINGITIS
E1060	23F	C51	VALLE	2006	PI-SXT-TET	MENINGITIS
E1075	23F	C51	VALLE	2006	PI-SXT-TET	MENINGITIS
3864	23F	C40	ANTIOQUIA	2007	PI-SXT	F. SYNDROME
3934	19F	C48	ANTIOQUIA	2007	PI-SXT	PNEUMONIA
E1672	23F	C55	BOGOTÁ	2010	PI-SXT-E-TET	MENINGITIS

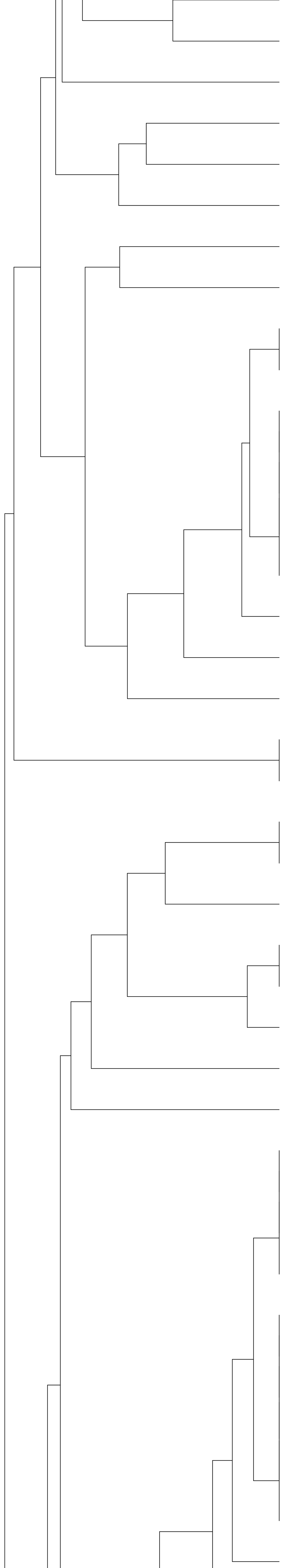

E1072	23F	C55	BOGOTÁ	2010	PI-SXT-E-TET	MENINGITIS
5170	23F	C50	BOLIVAR	2010	PI-SXT-TET	OTHER
E1090	15B	NR	BOGOTÁ	2006	PI-SXT	MENINGITIS
E945	9V	NR	ANTIOQUIA	2005	PH-CRO-SXT	PNEUMONIA
4867	23F	NR	BOLIVAR	2010	PI-SXT-E-TET	MENINGITIS
3245	15C	NR	BOGOTÁ	2006	PI-SXT	MENINGITIS
3364	10A	NR	VALLE	2006	PI-TET	MENINGITIS
E1369	NST	NR	BOGOTÁ	2008	PI-SXT	PNEUMONIA
E1689	19A	E1	ANTIOQUIA	2010	PH-CRO-SXT-E-TET	PNEUMONIA
4283	19A	E1-ST320	BOGOTÁ	2008	PH-CRO-SXT-E-TET	SEPSIS
E1631	19A	E-ST320	ANTIOQUIA	2010	PH-CRO-SXT-E-TET	PNEUMONIA
E1632	19A	E	ANTIOQUIA	2010	PH-CRO-SXT-E-TET	SEPSIS
E1650	19A	E	ANTIOQUIA	2010	PH-CRO-SXT-E-TET	BACTERIEMIA
5127	19A	E	ANTIOQUIA	2010	PH-CRO-SXT-E-TET	PNEUMONIA
E1688	19A	E	ANTIOQUIA	2010	PH-CRO-SXT-E-TET	SEPSIS
4793	19F	E2	BOGOTÁ	2009	PH-CRO-SXT-E-TET	SEPSIS
E1498	19A	E3	BOGOTÁ	2009	PH-CRO-SXT-E-TET	PNEUMONIA
2946	19F	E5	VALLE	2005	PH-SXT-E-TET	PNEUMONIA
3931	23F	NR	ANTIOQUIA	2007	PI-SXT	PNEUMONIA
3932	23F	NR	ANTIOQUIA	2007	PI-SXT	PNEUMONIA
4104	19A	F	ANTIOQUIA	2007	PI	PNEUMONIA
E1307	19A	F-ST1118	ANTIOQUIA	2007	PI	MENINGITIS
4654	6C	F	PNEUMONIA	2009 .	PI	
E1596	19A	F-ST118	ATLANTICO	2010	PI	F. SYNDROME
5112	19A	F	BOGOTÁ	2010	PI	PNEUMONIA
E1067	19A	F	ANTIOQUIA	2006	PI	SEPSIS
E1710	19A	F	BOGOTÁ	2010	PI	OTHER
4218	35B	NR	BOGOTÁ	2008	PH	SEPSIS
E1013	23F	A	VALLE	2006	PH-CRO-SXT-C-TET	MENINGITIS
E1038	23F	A	BOGOTÁ	2006	PH-CRO-SXT-C-TET	MENINGITIS
3500	23F	A	VALLE	2006	PH-CRO-SXT-C-TET	MENINGITIS
Spain23FST81	23F	A				
3952	23F	A3	BOGOTÁ	2007	PH-SXT-C-TET	OTHER
2971	23F	A3	VALLE	2005	PH-CRO-SXT-C-E-TET	SEPSIS
3030	23F	A3	BOGOTÁ	2005	PH-SXT-C-TET	BACTERIEMIA
4895	23F	A3	VALLE	2010	PH-CRO-SXT-C-TET	MENINGITIS
E1379	23F	A3	BOGOTÁ	2008	PH-SXT-C-TET	SEPSIS
E1458	23F	A3	BOGOTÁ	2008	PI-SXT-C-TET	SEPSIS
4397	23F	A14	ANTIOQUIA	2008	PH-CRO-SXT-C-TET	MENINGITIS

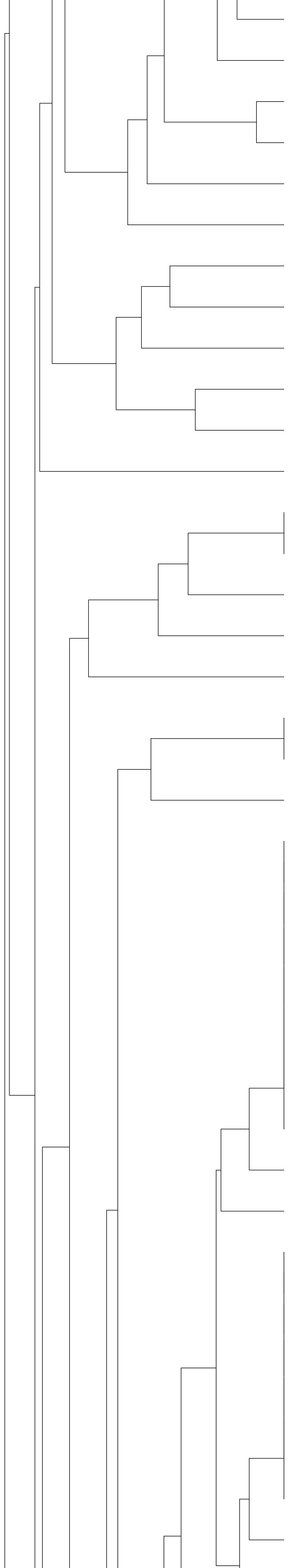

4397	23F	A14	ANTIOQUIA	2008	PH-CRO-SXT-C-TET	MENINGITIS
E1274	23F	A2	BOGOTÁ	2007	PH-CRO-SXT-C-E-TET	MENINGITIS
3953	23F	A11	BOGOTÁ	2007	PH-SXT-C-TET	PNEUMONIA
3459	19F	A12	VALLE	2006	PH-SXT-E	OTHER
4451	19A	A10- ST1.	META	2008	PI-E	MENINGITIS
5196	15A	NR	ATLANTICO	2010	PI	F. SYNDROME
E1216	14	NR	VALLE	2007	PI	F. SYNDROME
E1116	19A	NR	BOGOTÁ	2006	PI-SXT	SEPSIS
4471	19A	NR	BOGOTÁ	2008	PI-SXT	SEPSIS
3416	34	NR	CALDAS	2006	PI-SXT	MENINGITIS
4892	34	NR	RISARALDA	2010	PI-SXT	PNEUMONIA
4989	9N	NR	BOGOTÁ	2010	PI	MENINGITIS
E936	6B	NR	BOGOTÁ	2005	PI-SXT-C-E-TET	MENINGITIS
4564	6B	NR	BOGOTÁ	2009	PI-SXT-C-E-TET	PNEUMONIA
4894	6C	NR	PNEUMONIAE	2010 V.	PI	PNEUMONIA
4393	6B	NR	CESAR	2008	PI-SXT-C-E-TET	OTHER
4896	6C	NR	MENINGITIS	2010 .	PI	MENINGITIS
3363	23F	NR	ANTIOQUIA	2006	PI-SXT	SEPSIS
E997	6B	NR	SANTANDER	2006	PH-CRO-SXT-TET	MENINGITIS
3947	23F	NR	VALLE	2007	PH-CRO-SXT-C-E-TET	SEPSIS
E1180	6B	D8	CESAR	2007	PH-CRO-SXT	MENINGITIS
3915	6B	D8	BOGOTÁ	2007	PH-SXT-TET	OTHER
3487	6B	D8	BOGOTÁ	2006	PH-CRO-SXT-TET	MENINGITIS
4165	6B	D8	BOGOTÁ	2008	PH-CRO-SXT-TET	SEPSIS
E1138	6B	D8	BOGOTÁ	2006	PH-SXT-TET	BACTERIEMIA
E1468	6B	D8	CAQUETA	2009	PH-SXT-TET	MENINGITIS
E1471	6B	D8	BOGOTÁ	2009	PH-CRO-SXT-C-E-TET	PNEUMONIA
4128	6B	D8	SANTANDER	2008	PI-SXT-TET	SEPSIS
3274	6B	D14	SANTANDER	2006	PH-SXT-C-E	SEPSIS
E996	6B	D13	BOGOTÁ	2006	PH-SXT-TET	PNEUMONIA
E1172	6B	D	BOLIVAR	2007	PH-SXT-C-E-TET	PNEUMONIA
E1200	6B	D	BOGOTÁ	2007	PH-SXT-C-TET	PNEUMONIA
Spain6B-ST90	6B	D				
2941	6B	D	ANTIOQUIA	2005	PH-SXT-C-E	PNEUMONIA
3025	6B	D	TOLIMA	2005	PH-CRO-SXT-C-E-TET	MENINGITIS
5241	6B	D	ANTIOQUIA	2010	PH-SXT-C-E	SEPSIS
E1339	6B	D	BOGOTÁ	2008	PH-SXT-C-E	PNEUMONIA
5059	6B	D15	ANTIOQUIA	2010	PH-SXT-C-TET	PNEUMONIA

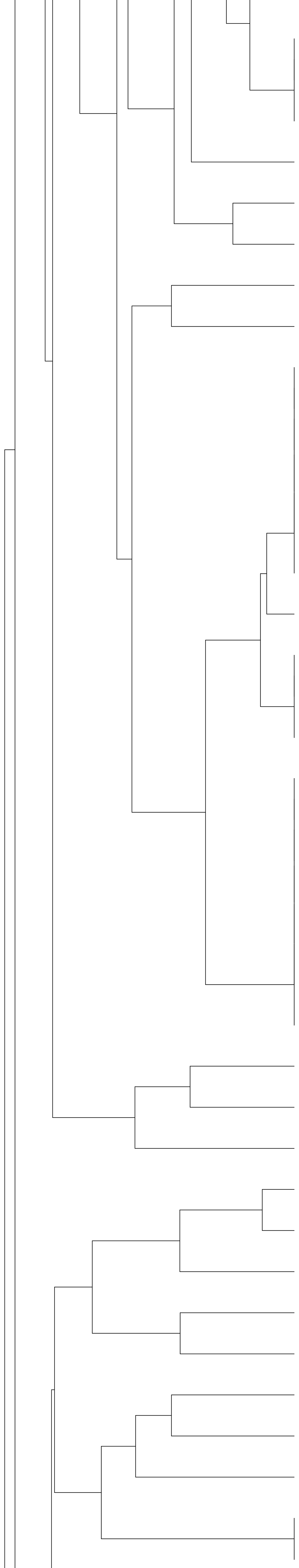

[illegible]	6B	D1[illegible]	ANTIOQUIA	2010	[illegible]	PNEUMONIA
3898	6B	D3	ANTIOQUIA	2007	PH-SXT-C-E-TET	PNEUMONIA
E892	6B	D3	BOGOTÁ	2005	PH-SXT-C-E-TET	PNEUMONIA
5087	6B	D3	SANTANDER	2010	PH-CRO-SXT-C-E-TET	SEPSIS
3411	6B	D12	VALLE	2006	PH-SXT-C-E	PNEUMONIA
3859	6B	D18	ANTIOQUIA	2007	PH-SXT-C-E	PNEUMONIA
E1251	6B	D16	VALLE	2007	PH-SXT-C-E-TET	SEPSIS
E947	14	K2	BOLIVAS	2005	PI-SXT-TET	PNEUMONIA
3267	6B	NR	VALLE	2006	PI-E	PNEUMONIA
E915	14	K3	ANTIOQUIA	2005	PI-TET	PNEUMONIA
E1485	14	K3	BOLIVAR	2009	PI-SXT-TET	MENINIGITIS
4682	15A	K3	BOLIVAR	2009	PI-E-TET	PNEUMONIA
4749	14	K3	ANTIOQUIA	2009	PI-SXT-TET	MENINGITIS
E1410	14	K3	VALLE	2008	PI	MENINGITIS
E1452	14	K3	VALLE	2008	PI	MENINGITIS
5126	15F	K6	ANTIOQUIA	2010	PH-CRO-E-TET	MENINGITIS
E1072	19F	K4	VALLE	2006	PI-E-TET	SEPSIS
3993	15A	K4	BOGOTÁ	2007	PI-E-TET	PNEUMONIA
5155	15A	K4	ANTIOQUIA	2010	PI-E-TET	F. SYNDROME
E995	14	K1	ARAUCA	2006	PI-SXT-TET	MENINGITIS
5005	14	K1	ANTIOQUIA	2010	PI-SXT-TET	PNEUMONIA
E1660	14	K1	CALDAS	2010	PI-SXT-TET	PNEUMONIA
E1671	14	K1	BOGOTÁ	2010	PH-SXT	PNEUMONIA
5064	14	K1	BOLIVAR	2010	PI-SXT-TET	MENINGITIS
4282	14	K1	BOGOTÁ	2008	PI-SXT-TET	SEPSIS
E1263	14	K1	CALDAS	2007	PI-SXT-TET	MENINGITIS
E1507	19F	NR	BOGOTÁ	2009	PI	SEPSIS
E1580	6B	NR	BOGOTÁ	2010	PI-SXT	PNEUMONIA
E1609	6B	NR	CORDOBA	2010	PI-SXT-TET	MENINGITIS
E1122	6B	NR	VALLE	2006	PI-SXT	SEPSIS
E1074	6B	NR	VALLE	2006	PI-SXT	SEPSIS
4690	6B	NR	CALDAS	2009	PI-SXT	MENINGITIS
E1587	6B	NR	SANTANDER	2010	PI-SXT-TET	MENINGITIS
E1706	11A	NR	VALLE	2010	PI	MENINGITIS
4243	19F	NR	BOGOTÁ	2008	PH-SXT-E-TET	MENINGITIS
E1414	19F	NR	BOGOTÁ	2008	PH-SXT	PNEUMONIA
E985	19F	NR	BOGOTÁ	2006	PI-SXT-TET	SEPSIS
E1595	19A	NR	BOGOTÁ	2010	PI	SEPSIS
5116	19A	NR	BOGOTÁ	2010	PI-E	SEPSIS

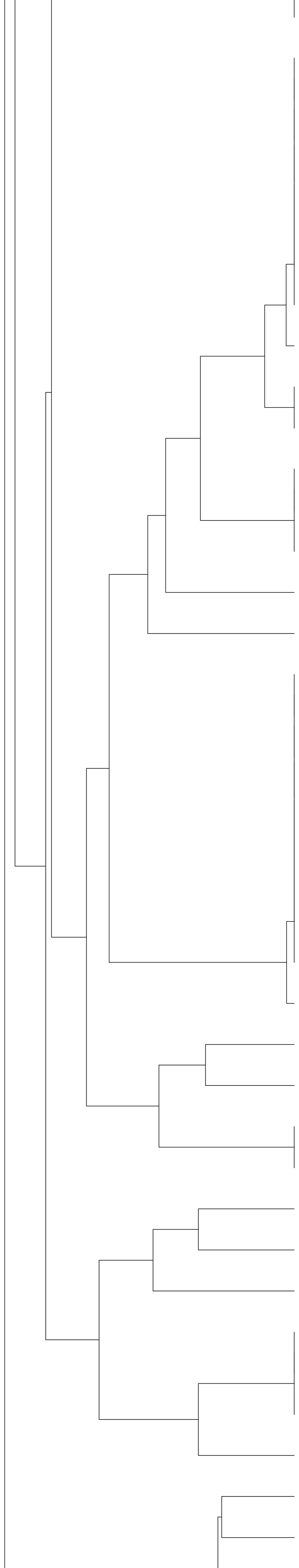

5116	19A	NR	BOGOTÁ	2010	PI-E	SEPSIS
3431	6A	I	BOGOTÁ	2006	PI-SXT	SEPSIS
E988	6A	I	BOGOTÁ	2005	PI-SXT	PNEUMONIA
E1483	6A	I-ST460	BOGOTÁ	2009	PI-SXT	PNEUMONIA
4776	6A	I	CASANARE	2009	PI-SXT	MENINGITIS
4646	6A	I	BOGOTÁ	2009	PI-SXT	SEPSIS
5163	6A	I	BOGOTÁ	2010	PI-SXT	F. SYNDROME
E1199	6A	I	BOGOTÁ	2007	PI-SXT	OTHER
4566	6A	I	BOGOTÁ	2009	PI	MENINGITIS
E1190	14	I1	BOGOTÁ	2007	PH-SXT	PNEUMONIA
E1541	14	I1	ANTIOQUIA	2009	PI-SXT-TET	MENINGITIS
3407	18A	I2	CALDAS	2006	PI-SXT-TET	PNEUMONIA
4864	12F	I2	VALLE	2010	PI	F. SYNDROME
5003	18A	I2	ANTIOQUIA	2010	PI-SXT-TET	BACTERIEMIA
E1579	6A	I3	BOGOTÁ	2010	PI-SXT	PNEUMONIA
E1001	6A	I5	BOYACA	2006	PH-CRO-SXT-TET	MENINGITIS
4001	14	J	ANTIOQUIA	2007	PI-SXT-TET	SEPSIS
E1237	14	J	ANTIOQUIA	2007	PI-SXT	SEPSIS
E975	14	J	VALLE	2005	PI-SXT-TET	F. SYNDROME
E1528	14	J	RISARALDA	2009	PI-SXT-TET	PNEUMONIA
E1562	14	J	ANTIOQUIA	2009	PI-SXT-TET	SEPSIS
4732	14	J	ANTIOQUIA	2009	PI-SXT-TET	PNEUMONIA
E-1687	14	J	ANTIOQUIA	2010	PH-SXT-TET	F. SYNDROME
3038	14	J	ANTIOQUIA	2005	PI-SXT-TET	PNEUMONIA
3263	14	J	ANTIOQUIA	2006	PI-SXT-TET	SEPSIS
4960	7F	NR	CALDAS	2010	PI-SXT	MENINGITIS
E1306	35B	NR	VALLE	2008	PI	MENINGITIS
E973	14	NR	ANTIOQUIA	2005	PH-SXT-TET	PNEUMONIA
E1012	23F	NR	VALLE	2006	PH-SXT-C-TET	MENINGITIS
E1310	6B	NR	CESAR	2007	PI-SXT-C-E-TET	MENINGITIS
E879	14	NR	ANTIOQUIA	2005	PI-SXT-TET	PNEUMONIA
4890	19F	NR	BOGOTÁ	2010	PH-CRO-SXT-C-E-TET	SEPSIS
4579	18C	NR	HUILA	2009	PI	MENINGITIS
4876	18C	NR	ANTIOQUIA	2010	PI	PNEUMONIA
4911	18C	NR	BOGOTÁ	2010	PI	F. SYNDROME
4609	18F	NR	BOGOTÁ	2009	PI-SXT	PNEUMONIA
E1236	14	B62	BOGOTÁ	2007	PH-SXT	PNEUMONIA
E1683	14	B101	ANTIOQUIA	2010	PH-CRO-SXT	SEPSIS
[illegible]	[illegible]	[illegible]	[illegible]	[illegible]	[illegible]	[illegible]

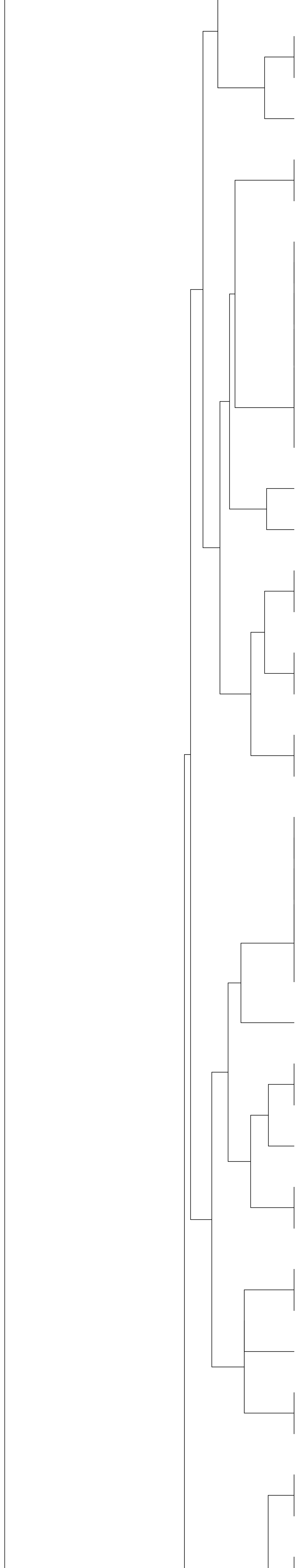

[illegible]	[illegible]	[illegible]	[illegible]	[illegible]	[illegible]	[illegible]
3836	14	B17	BOGOTÁ	2007	PH-CRO-SXT	PNEUMONIA
5202	14	B17	CALDAS	2010	PH-SXT	BACTERIEMIA
4840	14	B122	VALLE	2009	PH-CRO-SXT	PNEUMONIA
E1370	14	B86	ANTIOQUIA	2008	PH-SXT	PNEUMONIA
E1649	14	B86	BOGOTÁ	2010	PH-SXT	PNEUMONIA
E1242	14	B61	SANTANDER	2007	PH-SXT	SEPSIS
4379	14	B61	BOGOTÁ	2008	PH-CRO-SXT	PNEUMONIA
4527	14	B61	BOYACA	2009	PH-CRO-SXT	MENINIGITIS
E1605	14	B61	BOGOTÁ	2010	PH-SXT	PNEUMONIA
5237	14	B61	RISARALDA	2010	PH-SXT	PNEUMONIA
E1465	14	B61	BOGOTÁ	2008	PH-CRO-SXT	PNEUMONIA
E1257	14	B79	BOGOTÁ	2007	PH-CRO-SXT	PNEUMONIA
E1230	14	B80	BOGOTÁ	2007	PH-CRO-SXT	SEPSIS
3572	14	B42	VALLE	2006	PH-SXT	PNEUMONIA
3442	14	B42	VALLE	2006	PH-SXT	SEPSIS
E1347	14	B77	VALLE	2008	PH-CRO-SXT-E-TET	SEPSIS
E1486	14	B77	CALDAS	2009	PH-SXT	SEPSIS
E1149	14	B45	VALLE	2007	PH-CRO-SXT	MENINGITIS
E1338	14	B45	BOGOTÁ	2008	PI-SXT	PNEUMONIA
3345	9V	B28	ANTIOQUIA	2006	PH-SXT	PNEUMONIA
3356	14	B28	BOGOTÁ	2006	PH-SXT	SEPSIS
E1107	9V	B28	VALLE	2006	PH-SXT	PNEUMONIA
E1011	14	B28	BOGOTÁ	2006	PH-CRO-SXT	MENINGITIS
E1020	14	B28	BOGOTÁ	2006	PH-SXT	PNEUMONIA
3358	9V	B96	BOGOTÁ	2006	PH-SXT	SEPSIS
4881	14	B	ANTIOQUIA	2010	PH-CRO-SXT	PNEUMONIA
Spain9VST156	9V	B			P-CRO-STX	
E989	9V	B99	VALLE	2005	PI-TET	MENINGITIS
3948	9V	B68	BOGOTÁ	2007	PH-SXT-E	SEPSIS
3139	9V	B68	BOGOTÁ	2005	PH-CRO-SXT	MENINGITIS
4028	14	B55	BOGOTÁ	2007	PH-CRO-SXT	PNEUMONIA
E1173	14	B55	BOGOTÁ	2007	PH-SXT	PNEUMONIA
E999	14	B58	BOGOTÁ	2006	PH-SXT	PNEUMONIA
E1293	14	B60	ANTIOQUIA	2007	PH-SXT	SEPSIS
E1540	14	B60	ANTIOQUIA	2009	PH-CRO-SXT	SEPSIS
4697	14	B71	BOGOTÁ	2009	PH-SXT	SEPSIS
5235	14	B71	BOGOTÁ	2010	PH-SXT	PNEUMONIA
E1245	14	B36	BOGOTÁ	2007	PH-CRO-SXT	SEPSIS

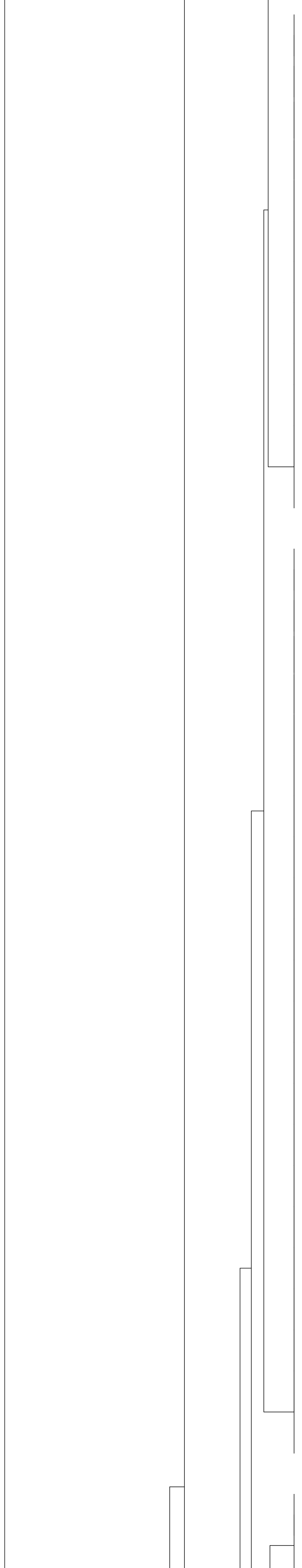

E1245	14	B36	BOGOTÁ	2007	PH-CRO-SXT	SEPSIS
3756	14	B36	BOGOTÁ	2007	PH-SXT	PNEUMONIA
E934	14	B36	BOGOTÁ	2005	PH-SXT	F. SYNDROME
E1378	14	B36	BOGOTÁ	2008	PH-CRO-SXT	PNEUMONIA
E1034	14	B36	BOGOTÁ	2006	PH-SXT	PNEUMONIA
E1406	14	B36	BOGOTÁ	2008	PH-SXT	SEPSIS
4554	14	B36	BOGOTÁ	2009	PH-CRO-SXT-C	PNEUMONIA
E1470	14	B36	BOGOTÁ	2009	PH-CRO-SXT	PNEUMONIA
E-1504	14	B36	HUILA	2009	PH-CRO-SXT-C	SEPSIS
4778	14	B36	NARIÑO	2009	PH-CRO-SXT-E	MENINGITIS
4802	14	B36	BOGOTÁ	2009	PI-CRO-SXT	MENINGITIS
E-1591	14	B36	BOGOTÁ	2010	PH-CRO-SXT	PNEUMONIA
3092	14	B36	TOLIMA	2005	PH-SXT	OTHER
E1192	14	B29	BOGOTÁ	2007	PH-SXT	PNEUMONIA
E1182	14	B29	BOYACA	2007	PH-CRO-SXT	MENINIGITIS
E1186	14	B29	BOGOTÁ	2007	PH-SXT	PNEUMONIA
E1336	14	B29	BOGOTÁ	2008	PH-SXT	BACTERIEMIA
E1352	14	B29	BOGOTÁ	2008	PH-CRO-SXT	SEPSIS
E1354	14	B29	BOGOTÁ	2008	PH-CRO-SXT	SEPSIS
E1359	14	B29	BOGOTÁ	2008	PH-CRO-SXT	MENINIGITIS
E1324	14	B29	BOGOTÁ	2008	PH-CRO-SXT	SEPSIS
4146	14	B29	BOGOTÁ	2008	PH-SXT	SEPSIS
E1422	14	B29	BOGOTÁ	2008	PH-SXT	PNEUMONIA
E1349	14	B29	BOGOTÁ	2008	PH-CRO-SXT	SEPSIS
E1465	14	B29	BOGOTÁ	2009	PH-CRO-SXT	PNEUMONIA
E1472	14	B29	BOGOTÁ	2009	PH-CRO-SXT	SEPSIS
E1469	14	B29	BOGOTÁ	2009	PH-CRO-SXT	PNEUMONIA
E1509	14	B29	BOGOTÁ	2009	PH-CRO-SXT	SEPSIS
4643	14	B29	BOGOTÁ	2009	PH-CRO-SXT	SEPSIS
E1608	14	B29	BOGOTÁ	2010	PH-CRO-SXT	PNEUMONIA
E1616	14	B29	BOGOTÁ	2010	PH-CRO-SXT	PNEUMONIA
5085	14	B29	BOGOTÁ	2010	PH-CRO-SXT	PNEUMONIA
5231	14	B29	BOGOTÁ	2010	PH-CRO-SXT-E-TET	MENINIGITIS
5230	14	B29	BOGOTÁ	2010	PH-CRO-SXT	MENINIGITIS
4365	14	B29	BOGOTÁ	2008	PI-SXT	PNEUMONIA
E872	14	B29	BOGOTÁ	2005	PH-CRO-SXT	MENINIGITIS
E1026	14	B104	BOGOTÁ	2006	PH-CRO-SXT	MENINIGITIS
E953	14	B104	BOGOTÁ	2005	PH-SXT	BACTERIEMIA
[illegible]	14	B104	BOGOTÁ	[illegible]	PH-SXT	PNEUMONIA

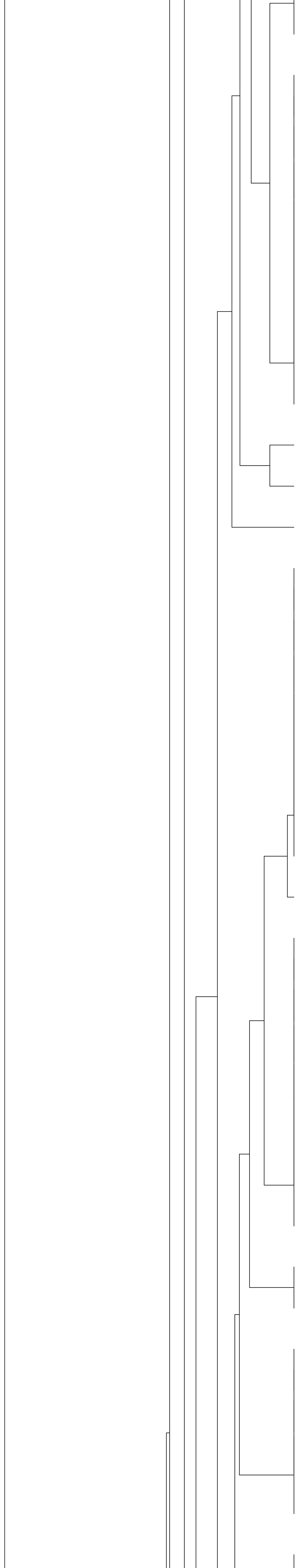

E1039	14	B104	BOGOTÁ	2006	PH-SXT	PNEUMONIA
3977	14	B53	BOGOTÁ	2007	PH-SXT	SEPSIS
E1279	14	B53	BOGOTÁ	2007	PH-CRO-SXT	PNEUMONIA
4221	14	B53	BOGOTÁ	2008	PH-CRO-SXT	SEPSIS
E1427	14	B53	BOGOTÁ	2008	PH-CRO-SXT	PNEUMONIA
E1412	14	B53	BOGOTÁ	2008	PH-CRO-SXT	PNEUMONIA
E1019	14	B53	BOGOTÁ	2006	PH-CRO-SXT	MENINIGITIS
E1104	14	B53	BOGOTÁ	2006	PH-SXT	SEPSIS
4669	14	B53	BOLIVAR	2009	PH-CRO-SXT	OTHER
4865	14	B53	TOLIMA	2010	PH-CRO-SXT	MENINIGITIS
4502	14	B103	BOGOTÁ	2009	PH-SXT	PNEUMONIA
E1029	14	B67	BOGOTÁ	2006	PH-SXT	OTHER
E1602	14	B129	RISARALDA	2010	PH-CRO-SXT	MENINIGITIS
E1174	14	B25	GUAJIRA	2007	PH-SXT	PNEUMONIA
4071	14	B25	VALLE	2008	PH-SXT	PNEUMONIA
E976	14	B25	BOGOTÁ	2005	PH-SXT	PNEUMONIA
E1473	14	B25	BOGOTÁ	2009	PH-CRO-SXT	PNEUMONIA
E-1500	14	B25	BOGOTÁ	2009	PH-CRO-SXT-C	PNEUMONIA
E-1462	9V	B25	BOGOTÁ	2009	PH-CRO-SXT	PNEUMONIA
4510	9V	B25	ATLANTICO	2009	PH-CRO-SXT	MENINIGITIS
4782	14	B25	SANTANDER	2009	PI-SXT	SEPSIS
5099	14	B25	ANTIOQUIA	2010	PH-CRO-SXT	SEPSIS
E1221	14	B30	BOGOTÁ	2007	PH-CRO-SXT	MENINIGITIS
3958	9V	B30	ANTIOQUIA	2007	PI-SXT	MENINIGITIS
3032	9V	B30	ANTIOQUIA	2005	PH-CRO-SXT	MENINIGITIS
4129	9V	B30	VALLE	2008	PI-SXT	SEPSIS
3302	14	B30	BOGOTÁ	2006	PH-SXT	SEPSIS
3480	9V	B30	ANTIOQUIA	2006	PH-SXT	PNEUMONIA
5025	9V	B30	RISARALDA	2010	PH-SXT	PNEUMONIA
E886	14	B30	VALLE	2005	PH-SXT	PNEUMONIA
E1188	14	B106	BOGOTÁ	2007	PH-SXT	PNEUMONIA
4213	14	B106	BOGOTÁ	2008	PH-CRO-SXT	SEPSIS
4093	14	B56	VALLE	2008	PH-CRO-SXT	PNEUMONIA
E1372	14	B56	ANTIOQUIA	2008	PH-CRO-SXT	PNEUMONIA
E1598	19A	B56	ATLANTICO	2010	PH-SXT-E	PNEUMONIA
E1543	19A	ST156	ANTIOQUIA	2009	PH-SXT-E	SEPSIS
E1705	14	B56	ANTIOQUIA	2010	PH-SXT	SEPSIS
E1270	9V	B1	ANTIOQUIA	2007	PH-CRO-SXT	SEPSIS

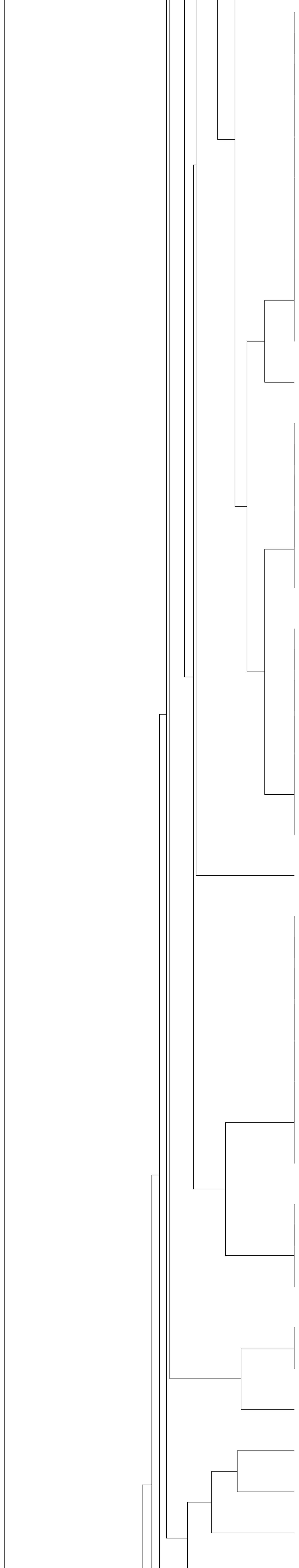

E1270	9V	B1	ANTIOQUIA	2007	PH-CRO-SXT	SEPSIS
E1206	14	B1	ANTIOQUIA	2007	PH-SXT	PNEUMONIA
E1238	14	B1	ANTIOQUIA	2007	PH-SXT	OTHER
4260	9V	B1	ANTIOQUIA	2008	PH-SXT	PNEUMONIA
E944	23F	B1	ATLANTICO	2005	PI-SXT-E-TET	PNEUMONIA
E1446	14	B1	BOGOTÁ	2008	PH-CRO-SXT	PNEUMONIA
4973	9V	B1	BOGOTÁ	2010	PH-SXT	F. SYNDROME
5052	9V	B1	ANTIOQUIA	2010	PH-SXT	PNEUMONIA
5222	9V	B1	SANTANDER	2010	PH-SXT	PNEUMONIA
3561	9V	B133	VALLE	2006	PI-SXT-TET	PNEUMONIA
3390	14	B43	BOGOTÁ	2006	PH-SXT	SEPSIS
E1082	14	B43	VALLE	2006	PH-SXT	SEPSIS
E1376	14	B43	BOGOTÁ	2008	PH-SXT-E	PNEUMONIA
E1600	14	B43	BOGOTÁ	2010	PH-CRO-SXT	PNEUMONIA
E1448	14	B43	BOGOTÁ	2008	PI-SXT	MENINIGITIS
3740	14	B16	ANTIOQUIA	2007	PH-SXT	PNEUMONIA
E1179	14	B16	BOGOTÁ	2007	PH-SXT	PNEUMONIA
E1639	14	B16	ANTIOQUIA	2010	PH-CRO-SXT	MENINIGITIS
5027	14	B16	CASANARE	2010	PH-SXT	SEPSIS
5130	14	B16	BOGOTÁ	2010	PH-SXT	PNEUMONIA
E-1680	14	B16	ANTIOQUIA	2010	PI-SXT-TET	SEPSIS
E1407	14	B78	BOGOTÁ	2008	PI-SXT	OTHER
E1094	14	B134	BOGOTÁ	2006	PH-CRO-SXT	SEPSIS
E1478	14	B134	BOGOTÁ	2009	PH-CRO-SXT	PNEUMONIA
E1495	14	B134	BOGOTÁ	2009	PH-SXT	F. SYNDROME
4635	14	B134	RISARALDA	2009	PH-CRO-SXT	PNEUMONIA
E1516	14	B134	BOGOTÁ	2009	PH-SXT	SEPSIS
E1582	14	B134	BOGOTÁ	2010	PH-CRO-SXT	PNEUMONIA
4885	14	B134	BOGOTÁ	2010	PH-CRO-SXT	SEPSIS
3353	14	B44	BOGOTÁ	2006	PH-SXT	PNEUMONIA
E1140	9V	B44	ANTIOQUIA	2006	PH-CRO-SXT	PNEUMONIA
E1003	14	B44	CALDAS	2006	PH-SXT	PNEUMONIA
E1099	14	B76	BOGOTÁ	2006	PH-CRO-SXT	MENINGITIS
E1093	14	B76	BOGOTÁ	2006	PH-SXT	SEPSIS
E1505	14	B108	BOGOTÁ	2009	PH-CRO-SXT	PNEUMONIA
4003	14	B63	ANTIOQUIA	2007	PH-CRO-SXT-E	F. SYNDROME
E1402	14	B98	BOGOTÁ	2008	PH-CRO-SXT	PNEUMONIA
4719	14	B81	BOGOTÁ	2009	PH-SXT	SEPSIS
[illegible]	[illegible]	[illegible]	[illegible]	[illegible]	[illegible]	[illegible]

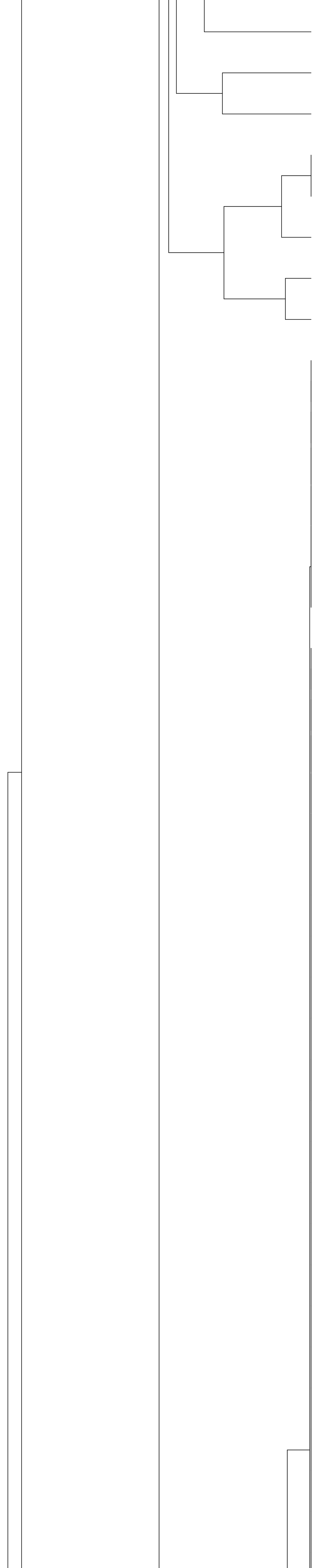

E-1502	14	B66	BOGOTÁ	2009	PH-CRO-SXT-C	F. SYNDROME
E873	14	B82	SANTANDER	2005	PH-SXT	SEPSIS
3247	14	B91	TOLIMA	2006	PH-CRO-SXT	MENINGITIS
3395	9V	B136	ANTIOQUIA	2006	PH-SXT	SEPSIS
3343	9V	B136	ANTIOQUIA	2006	PH-SXT	PNEUMONIA
E1079	9V	B139	VALLE	2006	PH-SXT	SEPSIS
E992	14	B135	BOGOTÁ	2005	PH-SXT	SEPSIS
3492	14	B132	BOGOTÁ	2006	PH-CRO-SXT	MENINGITIS
E1147	14	B3	VALLE	2007	PH-SXT-TET	F. SYNDROME
E1397	14	B3	VALLE	2008	PH-SXT-TET	OTHER
E960	14	B3	BOGOTÁ	2005	PH-CRO-SXT	BACTERIEMIA
E967	14	B3	VALLE	2005	PI-CRO-SXT	MENINGITIS
E971	14	B3	BOGOTÁ	2005	PH-SXT	PNEUMONIA
E994	14	B3	BOGOTÁ	2005	PH-CRO-SXT	MENINGITIS
E904	14	B3	BOGOTÁ	2005	PH-CRO-SXT	MENINGITIS
E1258	14	B3	ATLANTICO	2007	PH-SXT	PNEUMONIA
E1303	14	B3	BOGOTÁ	2007	PH-CRO-SXT	MENINGITIS
3738	14	B3	ATLANTICO	2007	PH-SXT	SEPSIS
3942	14	B3	BOGOTÁ	2007	PI-SXT	SEPSIS
3957	14	B3	ANTIOQUIA	2007	PI-SXT	PNEUMONIA
3961	14	B3	BOGOTÁ	2007	PH-CRO-SXT	PNEUMONIA
4000	14	B3	ANTIOQUIA	2007	PH-CRO-SXT	SEPSIS
E1198	14	B3	BOGOTÁ	2007	PH-SXT	PNEUMONIA
E1228	14	B3	BOGOTÁ	2007	PH-SXT	PNEUMONIA
E1205	14	B3	BOGOTÁ	2007	PH-SXT	PNEUMONIA
E1219	14	B3	BOGOTÁ	2007	PH-SXT	SEPSIS
E1223	14	B3	BOGOTÁ	2007	PH-SXT	PNEUMONIA
E1278	14	B3	BOGOTÁ	2007	PH-SXT	PNEUMONIA
E1155	14	B3	VALLE	2007	PI-SXT	PNEUMONIA
E1342	14	B3	BOGOTÁ	2008	PH-CRO-SXT	PNEUMONIA
E1360	14	B3	BOGOTÁ	2008	PH-SXT	PNEUMONIA
E1325	14	B3	BOGOTÁ	2008	PH-SXT	PNEUMONIA
4228	14	B3	BOGOTÁ	2008	PH-CRO-SXT	MENINGITIS
E1056	14	B3	BOGOTÁ	2006	PH-SXT	PNEUMONIA
3040	9V	B3	ANTIOQUIA	2005	PH-SXT	PNEUMONIA
E895	14	B3	VALLE	2005	PH-SXT	F. SYNDROME
E899	14	B3	BOGOTÁ	2005	PH-SXT	PNEUMONIA
E912	14	B3	BOGOTÁ	2005	PH-SXT	BACTERIEMIA

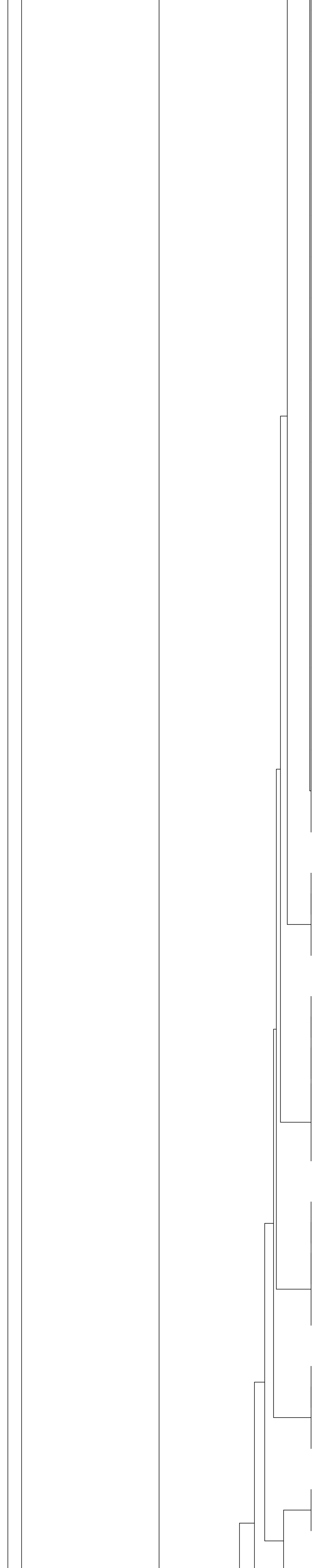

E912	14	B3	BOGOTÁ	2005	PH-SXT	BACTERIEMIA
E937	14	B3	BOGOTÁ	2005	PH-CRO-SXT	MENINGITIS
E941	14	B3	BOGOTÁ	2005	PI-SXT	PNEUMONIA
E942	14	B3	BOGOTÁ	2005	PI-SXT	F. SYNDROME
E1329	14	B3	BOGOTÁ	2008	PH-SXT	PNEUMONIA
E1383	14	B3	BOGOTÁ	2008	PI-SXT	PNEUMONIA
3951	14	B3	BOYACÁ	2007	PH-SXT	OTHER
E1068	14	B3	CALDAS	2006	PH-CRO-SXT	MENINGITIS
E1103	14	B3	BOGOTÁ	2006	PH-SXT	PNEUMONIA
E1531	14	B3	VALLE	2009	PH-SXT	PNEUMONIA
4744	14	B3	BOGOTÁ	2009	PI-SXT	SEPSIS
E1474	14	B3	BOGOTÁ	2009	PH-CRO-SXT	PNEUMONIA
E1522	14	B3	VALLE	2009	PH-SXT	OTHER
E1553	14	B3	BOYACÁ	2009	PH-SXT	SEPSIS
E1526	14	B3	BOGOTÁ	2009	PH-SXT	SEPSIS
4898	14	B3	VALLE	2010	PH-SXT	F. SYNDROME
4349	14	B3	BOGOTÁ	2008	PH-SXT	PNEUMONIA
2970	14	B3	BOGOTÁ	2005	PH-CRO-SXT	PNEUMONIA
3061	14	B3	BOGOTÁ	2005	PH-SXT	PNEUMONIA
E1438	14	B3	BOGOTÁ	2008	PH-SXT-E	F. SYNDROME
E1091	14	B3	BOGOTÁ	2006	PH-CRO-SXT	MENINGITIS
E948	14	B3	VALLE	2005	PI-SXT	PNEUMONIA
E1137	14	B3	BOGOTÁ	2006	PH-SXT	BACTERIEMIA
E954	14	B3	BOGOTÁ	2005	PI-SXT	BACTERIEMIA
3596	14	B39	BOGOTÁ	2007	PI-SXT	OTHER
E1350	14	B39	CASANARE	2008	PI-SXT	PNEUMONIA
E1320	14	B39	BOGOTÁ	2008	PH-CRO-SXT	MENINIGITIS
E1102	14	B39	BOGOTÁ	2006	PI-SXT	PNEUMONIA
E1601	14	B39	BOGOTÁ	2010	PH-CRO-SXT	PNEUMONIA
3366	14	B4	BOYACA	2006	PI-CRO-SXT	MENINGITIS
4380	14	B4	BOGOTÁ	2008	PH-SXT	PNEUMONIA
E1389	14	B4	BOGOTÁ	2008	PI-SXT	F. SYNDROME
E1440	14	B4	ANTIOQUIA	2008	PH-SXT-TET	PNEUMONIA
E1482	14	B88	BOGOTÁ	2009	PH-SXT	PNEUMONIA
E1492	14	B88	BOGOTÁ	2009	PH-CRO-SXT	F. SYNDROME
E1515	14	B88	BOGOTÁ	2009	PH-CRO-SXT	SEPSIS
E1340	14	B109	BOYACÁ	2008	PH-CRO-SXT	MENINGITIS
E1382	14	B109	BOGOTÁ	2008	PH-SXT	PNEUMONIA
4664	14	B120	BOGOTÁ	2009	PH-CRO-SXT	MENINGITIS

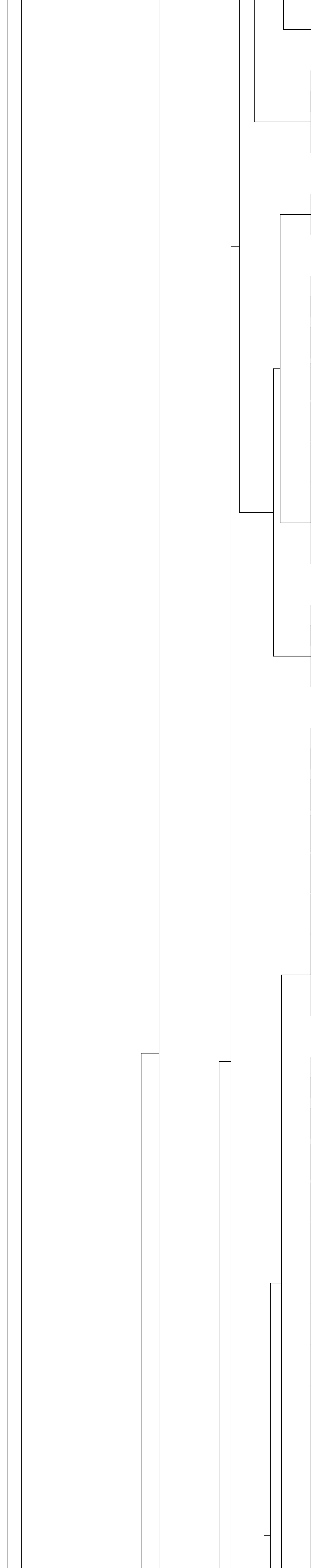

4664	14	B120	BOGOTÁ	2009	PH-CRO-SXT	MENINGITIS
E931	14	B73	VALLE	2005	PH-SXT-TET	F. SYNDROME
E935	14	B73	BOGOTÁ	2005	PH-SXT	PNEUMONIA
5156	14	B73	ANTIOQUIA	2010	PH-CRO-SXT	OTHER
E1004	14	B7	BOGOTÁ	2006	PI-SXT	PNEUMONIA
E1529	14	B7	SUCRE	2009	PH-SXT	PNEUMONIA
E1194	14	B18	BOGOTÁ	2007	PH-SXT	SEPSIS
E1154	14	B18	BOGOTÁ	2007	PH-SXT	BACTERIEMIA
E1043	14	B18	BOGOTÁ	2006	PH-CRO-SXT	MENINGITIS
E958	14	B18	ANTIOQUIA	2005	PI-SXT	MENINGITIS
E928	14	B18	CASANARE	2005	PH-SXT	SEPSIS
E1391	14	B18	BOGOTÁ	2008	PH-SXT	PNEUMONIA
3493	14	B18	BOGOTÁ	2006	PH-CRO-SXT	MENINGITIS
E1475	14	B18	SANTANDER	2009	PH-CRO-SXT	MENINGITIS
3140	9V	B83	BOGOTÁ	2005	PH-SXT	SEPSIS
2919	14	B83	VALLE	2005	PI-SXT-TET	PNEUMONIA
E916	14	B83	VALLE	2005	PH-SXT-TET	PNEUMONIA
E1330	14	B26	BOGOTÁ	2008	PH-SXT	PNEUMONIA
E888	14	B26	BOGOTÁ	2005	PH-SXT	PNEUMONIA
E1327	14	B26	BOGOTÁ	2008	PH-SXT	PNEUMONIA
E1415	14	B26	BOGOTÁ	2008	PH-CRO-SXT	OTHER
4264	14	B26	ANTIOQUIA	2008	PI-SXT	PNEUMONIA
E1403	14	B26	BOGOTÁ	2008	PH-SXT	PNEUMONIA
4928	14	B26	BOGOTÁ	2010	PH-SXT	SEPSIS
E-1691	14	B26	BOGOTÁ	2010	PH-CRO-SXT	F. SYNDROME
3430	14	B13	BOGOTÁ	2006	PH-SXT	SEPSIS
E1196	14	B13	BOGOTÁ	2007	PH-CRO-SXT	SEPSIS
3949	14	B13	BOGOTÁ	2007	PH-SXT	PNEUMONIA
E1312	14	B13	BOGOTÁ	2008	PI-SXT	SEPSIS
E955	14	B13	CALDAS	2005	PH-CRO-SXT	MENINGITIS
E968	14	B13	BOGOTÁ	2005	PH-SXT	SEPSIS
E905	14	B13	BOLIVAR	2005	PH-SXT	PNEUMONIA
E924	14	B13	BOGOTÁ	2005	PH-SXT-E	PNEUMONIA
E926	14	B13	BOGOTÁ	2005	PH-SXT	BACTERIEMIA
E1499	14	B13	BOGOTÁ	2009	PH-CRO-SXT-C	PNEUMONIA
E1488	14	B13	BOGOTÁ	2009	PH-SXT	PNEUMONIA
E1501	14	B13	BOGOTÁ	2009	PH-SXT-C	SEPSIS
5054	14	B13	RISARALDA	2010	PH-SXT	PNEUMONIA

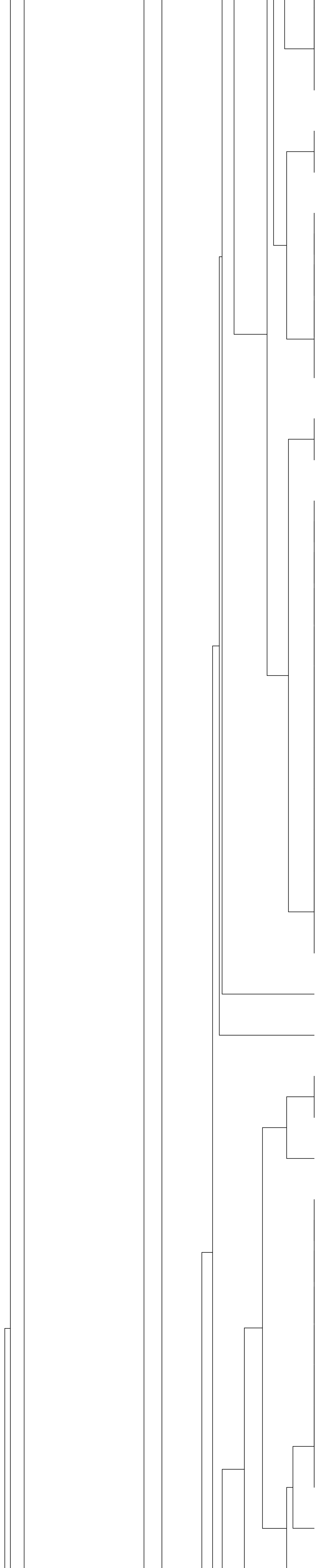

5054	14	B13	RISARALDA	2010	PH-SXT	PNEUMONIA
E1450	14	B13	BOGOTÁ	2008	PH-CRO-SXT	PNEUMONIA
E876	14	B13	ANTIOQUIA	2005	PI-SXT	PNEUMONIA
E1341	14	B50	BOGOTÁ	2008	PH-SXT	SEPSIS
E1344	14	B50	BOGOTÁ	2008	PH-SXT	SEPSIS
3518	14	B51	BOGOTÁ	2006	PH-CRO-SXT	PNEUMONIA
4259	14	B51	ANTIOQUIA	2008	PH-SXT	PNEUMONIA
3519	14	B51	BOGOTÁ	2006	PI-SXT-TET	PNEUMONIA
E1331	14	B51	BOGOTÁ	2008	PH-SXT	PNEUMONIA
E1642	14	B51	BOGOTÁ	2010	PH-SXT	SEPSIS
E1031	14	B52	BOGOTÁ	2006	PH-SXT	F. SYNDROME
E1493	14	B52	BOGOTÁ	2009	PH-CRO-SXT	PNEUMONIA
E1016	14	B10	BOYACÁ	2006	PH-CRO-SXT	MENINGITIS
E1184	14	B10	BOGOTÁ	2007	PH-CRO-SXT	MENINGITIS
E1161	14	B10	BOGOTÁ	2007	PI-SXT	MENINGITIS
E1164	14	B10	BOGOTÁ	2007	PH-SXT	PNEUMONIA
E1218	14	B10	BOGOTÁ	2007	PH-CRO-SXT	MENINIGITIS
E980	14	B10	BOGOTÁ	2005	PH-SXT	SEPSIS
E927	14	B10	VALLE	2005	PH-CRO-SXT	MENINGITIS
E932	14	B10	BOGOTÁ	2005	PH-SXT	PNEUMONIA
E1328	14	B10	BOGOTÁ	2008	PH-SXT	PNEUMONIA
E1429	14	B10	BOGOTÁ	2008	PH-SXT	PNEUMONIA
E1098	14	B10	BOGOTÁ	2006	PH-SXT	PNEUMONIA
E1484	14	B10	BOGOTÁ	2009	PH-SXT-E	PNEUMONIA
3198	14	B113	VALLE	2005	PH-SXT	SEPSIS
E949	14	B119	VALLE	2005	PH-SXT	OTHER
E891	14	B93	BOGOTÁ	2005	PH-SXT	SEPSIS
E1657	14	B93	BOGOTÁ	2010	PH-CRO-SXT	PNEUMONIA
E1322	14	B107	BOGOTÁ	2008	PH-SXT	PNEUMONIA
3781	14	B31	BOGOTÁ	2007	PH-SXT	PNEUMONIA
3439	14	B31	BOGOTÁ	2006	PH-SXT-TET	SEPSIS
E1288	14	B31	BOGOTÁ	2007	PH-CRO-SXT	MENINGITIS
E1169	14	B31	BOGOTÁ	2007	PI-SXT	PNEUMONIA
E919	14	B31	BOGOTÁ	2005	PH-CRO-SXT	MENINGITIS
E1063	14	B31	BOGOTÁ	2006	PH-CRO-SXT	MENINGITIS
E1521	14	B31	BOGOTÁ	2009	PH-CRO-SXT	SEPSIS
E1674	14	B31	CALDAS	2010	PH-SXT	PNEUMONIA
E943	14	B31	BOGOTÁ	2005	PI-SXT	PNEUMONIA
E1261	14	B116	BOGOTÁ	2007	PH-CRO-SXT	MENINGITIS

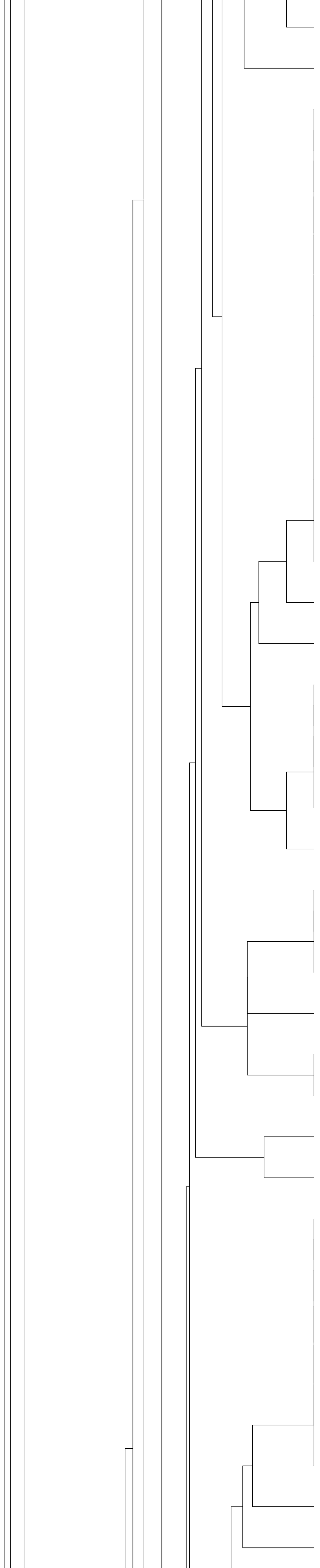

E1261	14	B116	BOGOTÁ	2007	PH-CRO-SXT	MENINGITIS
E1139	14	B117	BOGOTÁ	2006	PH-CRO-SXT	PNEUMONIA
3984	14	B27	ANTIOQUIA	2007	PH-SXT	OTHER
E1129	14	B27	VALLE	2006	PH-SXT	PNEUMONIA
E1042	14	B27	BOGOTÁ	2006	PH-SXT	SEPSIS
E894	14	B27	BOGOTÁ	2005	PH-SXT	PNEUMONIA
E898	14	B27	VALLE	2005	PH-SXT-TET	SEPSIS
E1506	14	B27	BOGOTÁ	2009	PH-CRO-SXT	SEPSIS
4667	14	B27	BOGOTÁ	2009	PH-SXT	SEPSIS
E880	14	B27	VALLE	2005	PH-SXT	SEPSIS
2973	14	B27	SANTANDER	2005	PH-SXT	SEPSIS
3212	14	B27	BOGOTÁ	2005	PH-SXT	PNEUMONIA
3240	14	B27	VALLE	2005	PH-SXT	SEPSIS
E1451	14	B27	BOGOTÁ	2008	PH-CRO-SXT	SEPSIS
5044	14	B102	ANTIOQUIA	2010	PH-SXT-E	PNEUMONIA
E979	14	B92	BOGOTÁ	2005	PH-SXT	PNEUMONIA
4018	14	B54	BOGOTÁ	2007	PI-SXT	SEPSIS
E1124	14	B54	VALLE	2006	PH-SXT-TET	F. SYNDROME
4281	14	B54	BOGOTÁ	2008	PH-SXT	PNEUMONIA
E1384	14	B54	BOGOTÁ	2008	PH-SXT	PNEUMONIA
E1548	9V	B128	ANTIOQUIA	2009	PH-SXT-E	PNEUMONIA
E1614	14	B69	BOGOTÁ	2010	PH-CRO-SXT	PNEUMONIA
E1663	14	B69	BOGOTÁ	2010	PH-CRO-SXT	PNEUMONIA
5239	14	B69	ANTIOQUIA	2010	PH-CRO-SXT	SEPSIS
E1248	14	B124	ANTIOQUIA	2007	PI-SXT	PNEUMONIA
E889	14	B95	BOGOTÁ	2005	PH-SXT	PNEUMONIA
4577	14	B95	SANTANDER	2009	PH-SXT	SEPSIS
E1153	14	B105	PUTUMAYO	2007	PH-CRO-SXT-E-TET	MENINGITIS
E982	14	B72	BOGOTÁ	2005	PH-CRO-SXT	MENINGITIS
E1226	14	B33	BOGOTÁ	2007	PH-SXT	PNEUMONIA
3736	14	B33	ANTIOQUIA	2007	PH-SXT	PNEUMONIA
E1211	14	B33	BOGOTÁ	2007	PH-CRO-SXT	PNEUMONIA
4242	14	B33	BOGOTÁ	2008	PH-SXT	PNEUMONIA
E1477	14	B33	BOGOTÁ	2009	PH-SXT	PNEUMONIA
4859	14	B33	BOGOTÁ	2009	PH-CRO-SXT	SEPSIS
4400	14	B33	ANTIOQUIA	2008	PH-SXT	PNEUMONIA
4853	14	B100	SANTANDER	2009	PH-CRO-SXT	MENINGITIS
E1062	14	B94	BOGOTÁ	2006	PH-SXT	SEPSIS

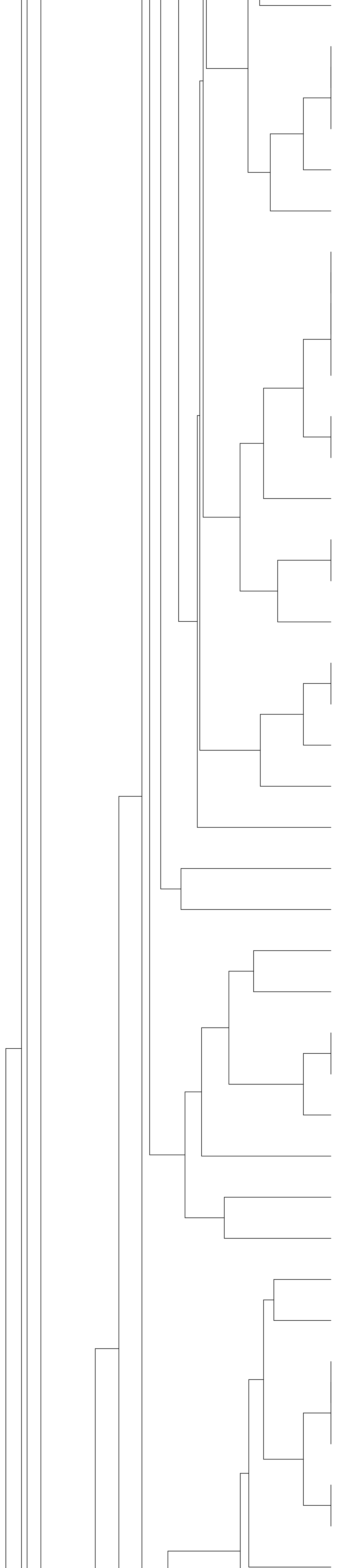

E1062	14	B94	BOGOTÁ	2006	PH-SXT	SEPSIS
E1323	14	B21	BOGOTÁ	2008	PH-SXT	PNEUMONIA
E900	14	B21	BOGOTÁ	2005	PH-SXT	PNEUMONIA
3304	14	B21	BOGOTÁ	2006	PI-SXT	SEPSIS
E1007	14	B118	BOGOTÁ	2006	PH-SXT	PNEUMONIA
4374	14	B87	BOYACA	2008	PH-SXT	OTHER
E1058	14	B85	BOGOTÁ	2006	PH-CRO-SXT	MENINGITIS
E1494	14	B85	BOGOTÁ	2009	PH-SXT	F. SYNDROME
E1503	14	B85	BOGOTÁ	2009	PH-SXT-C	F. SYNDROME
E1549	14	B85	CALDAS	2009	PH-CRO-SXT	OTHER
E1280	14	B20	BOGOTÁ	2007	PH-SXT	SEPSIS
E1142	14	B20	BOGOTÁ	2007	PH-SXT	SEPSIS
E1185	14	B65	BOYACA	2007	PH-CRO-SXT	MENINGITIS
E1715	14	B111	BOGOTÁ	2010	PH-SXT	SEPSIS
4492	14	B111	BOGOTÁ	2008	PH-SXT	PNEUMONIA
E974	14	B74	BOGOTÁ	2005	PH-SXT	PNEUMONIA
E1318	14	B59	BOGOTÁ	2008	PI-SXT	PNEUMONIA
3015	14	B59	CALDAS	2005	PH-CRO-SXT	MENINGITIS
E1225	14	B49	BOGOTÁ	2007	PH-SXT	F. SYNDROME
E1118	14	B89	VALLE	2006	PH-CRO-SXT	SEPSIS
4019	14	B136	BOGOTÁ	2007	PH-SXT	SEPSIS
E963	14	B83	BOGOTÁ	2005	PH-SXT	PNEUMONIA
E1381	14	B112	BOGOTÁ	2008	PH-SXT	PNEUMONIA
E1100	14	B123	BOGOTÁ	2006	PH-CRO-SXT	MENINGITIS
3288	9V	B130	CALDAS	2006	PH-SXT	MENINGITIS
E1527	14	B126	BOGOTÁ	2009	PH-CRO-SXT	SEPSIS
E1623	14	B126	BOGOTÁ	2010	PH-CRO-SXT	PNEUMONIA
E1141	14	B131	ANTIOQUIA	2006	PH-SXT	PNEUMONIA
3989	14	B137	BOGOTÁ	2007	PH-CRO-SXT	PNEUMONIA
E1337	14	B57	BOGOTÁ	2008	PH-SXT	PNEUMONIA
5109	18C	NR	BOGOTÁ	2010	PI	MENINGITIS
3861	9V	B136	ANTIOQUIA	2007	PH-CRO-SXT	PNEUMONIA
E951	14	B97	BOGOTÁ	2005	PH-CRO-SXT	PNEUMONIA
3215	14	B90	BOGOTÁ	2005	PH-SXT	PNEUMONIA
E911	14	B90	BOGOTÁ	2005	PH-SXT	PNEUMONIA
E1708	14	B90	BOGOTÁ	2010	PH-SXT	PNEUMONIA
E1146	14	B70	VALLE	2007	PH-CRO-SXT-E-TET	F. SYNDROME
3254	14	B70	VALLE	2006	PH-CRO-SXT-E-TET	PNEUMONIA
E1233	14	B75	BOGOTÁ	2007	PH-CRO-SXT	MENINGITIS

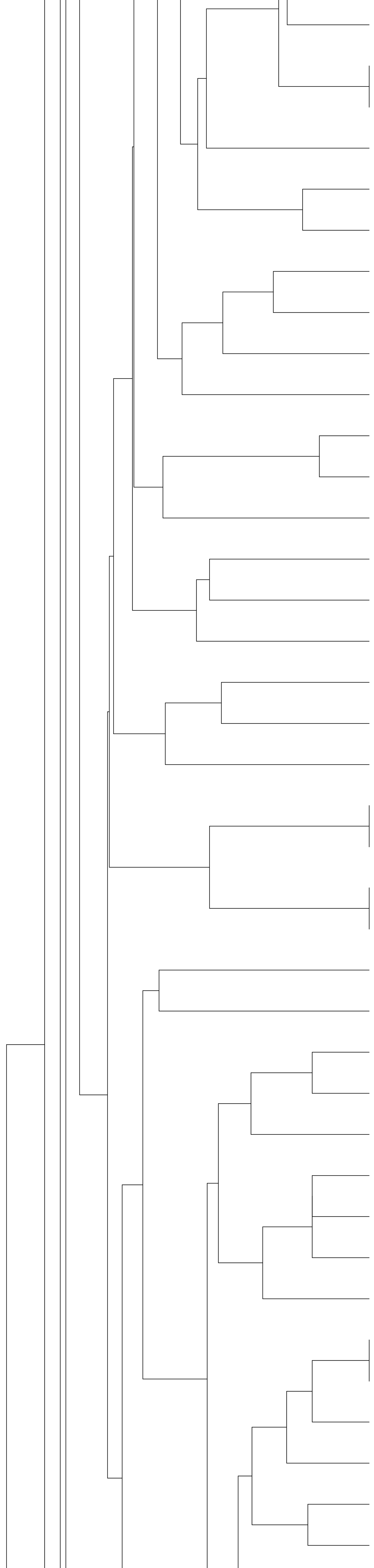

E1233	14	B75	BOGOTÁ	2007	PH-CRO-SXT	MENINGITIS
4967	9V	B47	RISARALDA	2010	PI-SXT	MENINGITIS
E1654	9V	B47	BOGOTÁ	2010	PH-SXT	F. SYNDROME
E1461	14	NR	BOGOTÁ	2009	PH-CRO-SXT	PNEUMONIA
E1224	14	NR	BOGOTÁ	2007	PH-SXT	SEPSIS
3327	14	B115	VALLE	2006	PH-SXT	SEPSIS
E1675	19A	NR	BOGOTÁ	2010	PH-CRO-SXT-E	PNEUMONIA
E1699	19A	NR	SANTANDER	2010	PI-SXT	PNEUMONIA
E1171	19A	NR	BOGOTÁ	2007	PH-CRO-SXT-E	PNEUMONIA
3334	23F	NR	VALLE	2006	PH-CRO-SXT	MENINGITIS
E952	14	B114	BOGOTÁ	2005	PH-CRO-SXT	MENINIGITIS
3008	14	B138	VALLE	2005	PH-CRO-SXT-TET	MENINGITIS
4988	6A	NR	ANTIOQUIA	2010	PI-E	SEPSIS
3049	25F	NR	ANTIOQUIA	2005	PI-SXT	PNEUMONIA
4309	14	NR	BOGOTÁ	2008	PH-CRO-SXT	MENINGITIS
E1301	6A	NR	SUCRE	2007	PI-E	PNEUMONIA
3965	14	NR	ANTIOQUIA	2007	PH-SXT	PNEUMONIA
E1110	23F	NR	NARIÑO	2006	PI-SXT-TET	MENINGITIS
E1635	9V	NR	BOGOTÁ	2010	PI-SXT	SEPSIS
E1431	14	NR	BOLIVAR	2008	PH-SXT	PNEUMONIA
E1432	14	NR	BOGOTÁ	2008	PH-SXT	SEPSIS
E1368	19F	NR	HUILA	2008	PI-SXT	MENINGITIS
E1433	19F	NR	HUILA	2008	PI	PNEUMONIA
4925	6A	NR	ATLANTICO	2010	PI-E	OTHER
E1677	6A	NR	ANTIOQUIA	2010	PI-E	SEPSIS
E1283	6A	H	ANTIOQUIA	2007	PI-E	MENINGITIS
4726	6A	H	ANTIOQUIA	2009	PI-E	SEPSIS
5012	6A	H	ANTIOQUIA	2010	PI-E	BACTERIEMIA
E1220	6A	H	BOGOTÁ	2007	PI-E	PNEUMONIA
4816	6A	H	ATLANTICO	2009	PI-E	MENINGITIS
5182	6A	H	TOLIMA	2010	PI-E	OTHER
4316	6A	H	VALLE	2008	PI-E	SEPSIS
E1335	6A	H	BOGOTÁ	2008	PI-E	PNEUMONIA
E1409	6A	H-ST473	BOGOTÁ	2008	PI-E	SEPSIS
4256	6A	H	SANTANDER	2008	PI-E	PNEUMONIA
3013	6A	H	SANTANDER	2005	PI-E	PNEUMONIA
3037	6A	H	ANTIOQUIA	2005	PI-E	PNEUMONIA
5228	9C	H	BOGOTÁ	2010	PI	MENINGITIS

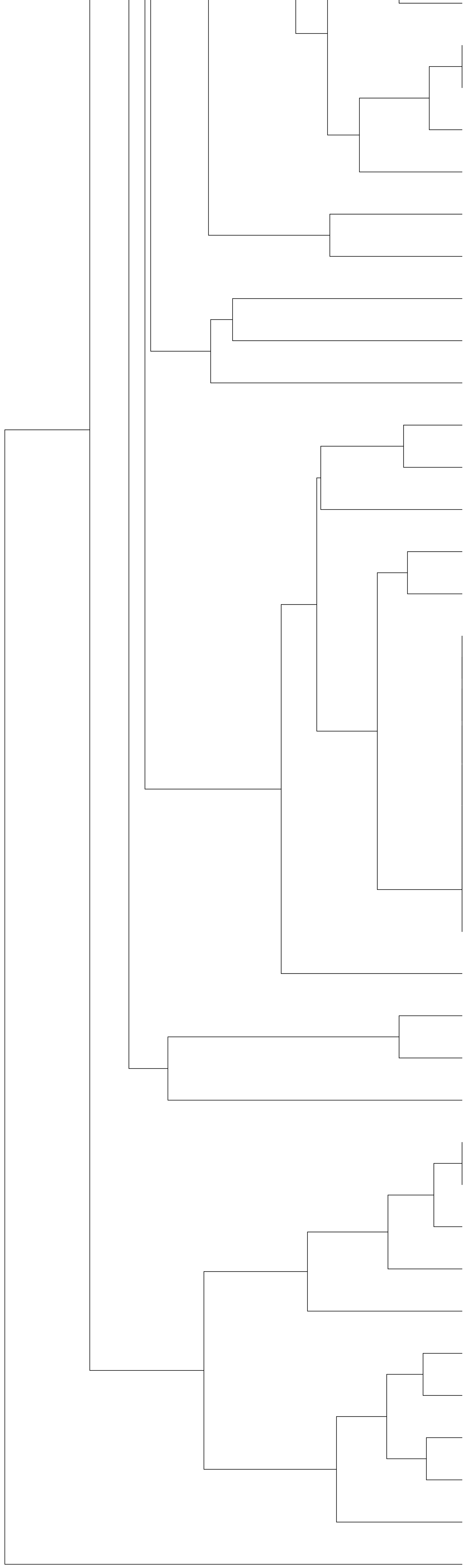

5228	9C	H	BOGOTÁ	2010	PI	MENINGITIS
3733	6A	H	ANTIOQUIA	2007	PI-E	MENINGITIS
E1195	6A	H	BOGOTÁ	2007	PI-E	SEPSIS
3830	6A	H	ANTIOQUIA	2007	PI-E	PNEUMONIA
3184	14	H	ANTIOQUIA	2005	PI-SXT	OTHER
3129	6B	NR	BOGOTÁ	2005	PI-SXT	OTHER
E1575	19A	NR	BOGOTÁ	2009	PI	SEPSIS
3197	19A	NR	CALDAS	2005	PI-SXT-TET	MENINGITIS
4866	19F	NR	BOGOTÁ	2010	PI	MENINGITIS
E1547	14	NR	ANTIOQUIA	2009	PI-SXT	SEPSIS
E1476	14	NR	BOGOTÁ	2009	PI-TET	PNEUMONIA
4983	19F	NR	CALDAS	2010	PH-CRO-SXT-E	MENINGITIS
E1604	14	NR	BOYACA	2010	PI-SXT-E-TET	PNEUMONIA
E918	19A	G	VALLE	2005	PI-SXT-E-TET	PNEUMONIA
E1599	19A	G	BOGOTÁ	2010	PH-SXT-E-TET	SEPSIS
E1250	19A	G	ANTIOQUIA	2007	PI-SXT-E-TET	F. SYNDROME
E1285	19A	G	ANTIOQUIA	2007	PH-SXT-E-TET	PNEUMONIA
E986	19A	G	ANTIOQUIA	2005	PH-SXT-E-TET	F. SYNDROME
E1467	19A	G	BOGOTÁ	2009	PH-CRO-SXT-E-TET	PNEUMONIA
4604	19A	G-ST276	BOGOTÁ	2009	PH-SXT-C-E-TET	SEPSIS
4735	19A	G	ANTIOQUIA	2009	PI-SXT-E-TET	SEPSIS
5134	19A	G	VALLE	2010	PI-CRO-SXT-E-TET	MENINIGITIS
5187	19A	G	ANTIOQUIA	2010	PH-CRO-SXT-E-TET	SEPSIS
3438	19F	NR	VALLE	2006	PH-SXT-E	SEPSIS
3048	6A	NR	ANTIOQUIA	2005	PI-E	MENINGITIS
3087	6A	NR	ANTIOQUIA	2005	PI-E	MENINGITIS
E-1593	23F	NR	BOGOTÁ	2010	PI	SEPSIS
E1109	19F	NR	BOGOTÁ	2006	PI-SXT-C-TET	MENINGITIS
3393	23F	NR	ANTIOQUIA	2006	PI-SXT	MENINGITIS
E1392	19F	NR	BOGOTÁ	2008	PI-SXT-C-E	PNEUMONIA
E1077	19F	NR	VALLE	2006	PI-SXT-C-TET	SEPSIS
4748	6A	NR	ANTIOQUIA	2009	PI-E	MENINGITIS
4150	6B	NR	BOGOTÁ	2008	PI-SXT-E-TET	PNEUMONIA
E1240	6B	NR	META	2007	PI-SXT-E	MENINGITIS
E1686	6B	NR	RISARALDA	2010	PI-SXT-E-TET	OTHER
E1693	6B	NR	ANTIOQUIA	2010	PI-SXT-E	F. SYNDROME
E1321	6B	NR	BOGOTÁ	2008	PI-SXT-E	SEPSIS
5062	NST	NR	BOGOTÁ	2010	PI	PNEUMONIA
