# Supplementary material for: Serotype and Genotype Distribution among Invasive Streptococcus pneumoniae Isolates in Colombia, 2005–2010
Source: PLoS One. 2014 Jan 8;9(1):e84993. doi: 10.1371/journal.pone.0084993 (PMC3885649; doi:10.1371/journal.pone.0084993)
Supplement: Table S3 — Susceptibility and minimum inhibitory concentrations in S. pneumoniae isolates in invasive pneumococcal diseases during 2005 and 2010. (DOC) [file pone.0084993.s005.doc]

Supporting Information

Table S3. Susceptibility and minimum inhibitory concentrations in *S. pneumoniae* isolates in invasive pneumococcal diseases during 2005 and 2010.

| Antimicrobian | Susceptibility n (%) | | | MIC (mg/ml) | |
| --- | --- | --- | --- | --- | --- |
|  | S | I | R | 50 | 90 |
| Penicillin |  |  |  |  |  |
| Meningitis | 321(69.3) | 0 | 142(30.7) | 0.03 | 2 |
| Non-meningitis | 1194(91.0) | 113(8.6) | 5(0.4) | 0.03 | 2 |
| Ceftriaxone |  |  |  |  |  |
| Meningitis | 390(84.2) | 58(12.5) | 15(3.2) | 0.03 | 1 |
| Non-meningitis | 1201(91.5) | 105(8.0) | 6(0.5) | 0.03 | 1 |
| Chloramphenicol | 1709(96.2) | 0 | 66 (3.7) | 2 | 4 |
| Erythromycin | 1651(93.0) | 6 (0.3) | 118(6.6) | 0.06 | 0.125 |
| Sulfamethoxazole–  trimethoprim | 997(56.2) | 174 (9.8) | 604 (34.0) | 0.05 | 16 |
| Tetracycline | 1471(82.9) | 28 (1.6) | 276(15.5) | 0.25 | 8 |

S=sensible, I=Intermediate and R=resistant.

All isolates were susceptible to vancomycin.
